# A cross-sectional study of routine hematological and biochemical parameters with age- and sex-specific analyses in mid-to-high-altitude regions of southwestern China

**DOI:** 10.3389/fpubh.2026.1949654

**Published:** 2026-10-28

**Authors:** Jing Li, Yong Ji, Shanshan Zhuang, Jia Wang, Xiang Zhang, Yue Fan, Yuanyuan Zhou, Xingying Chen, Jian Lang, Ye Kuang, Lei Feng

**Affiliations:** 1 Yan’an Hospital Affiliated to Kunming Medical University, Kunming, China; 2 Key Laboratory of Cardiovascular Disease of Yunnan Province, Kunming, China; 3 Clinical Medicine Center for Cardiovascular Disease of Yunnan Province, Kunming, China

**Keywords:** age, biochemical indicators, complete blood count, mid-to-high-altitude areas, physical examination population, sex

## Abstract

**Background:**

Complete blood count (CBC) and biochemical parameters (liver function, renal function, Blood glucose, and lipid profile) are the most routinely used tests in clinical laboratory practice, and serve as critical evidence for disease diagnosis. This study aimed to characterize the distribution patterns of age- and sex-related differences in CBC and biochemical indicators, identify the age-related inflection points of these indicators, and analyze their correlation patterns across different sexes and age groups.

**Methods:**

A total of 83,945 individuals physical examination in mid-to-high-altitude areas of Yuxi City, southwestern China (1,500–1,800 m above sea level), were enrolled in this study. All subjects were stratified into seven age groups by sex. Age trend inflection plots, correlation analysis, and mediation models were applied to explore indicator trajectories, inter-association patterns, and the mediating role of triglyceride (TG) in the age–uric acid (UA) relationship.

**Results:**

After FDR correction, most hematological and biochemical parameters remained statistically significant in sex comparisons within each age group. For indicators with an absolute relative median difference |RMD| > 10%, total bilirubin (TBIL), direct bilirubin (DBIL), alanine aminotransferase (ALT), creatinine (CREA), and uric acid (UA) consistently exhibited clinically meaningful sex differences across all age groups. Most included CBC and biochemical indicators included in this study showed generally synchronous age trend in both male and female populations, the age inflection points were also generally concordant between sexes. However, four indicators alkaline phosphatase (ALP), globulin (GLB), triglyceride (TG), and uric acid (UA) showed pronounced sexual dimorphism in their age-dependent trajectories. The CBC indicators showed that the levels of red blood cell count (RBC), hemoglobin (Hb), and hematocrit (HCT) were higher in males from 18–25 to 56–65 years, but females surpassed males by the 66–75 years group. Sex-stratified correlation analysis revealed that in young males (18–25 years), lipid metabolism indicators had the strongest correlations with liver enzymes and liver enzyme ratios, with correlation coefficients ranging from 0.31 to 0.39; these correlations gradually attenuated with advancing age. In females, only transient moderate correlations of UA with TG and VLDL emerged during the perimenopausal period (36–45 years). After Bonferroni correction, all variable pairs with |r| > 0.3 remained statistically significant across all age groups in females, whereas some variable pairs in males failed to reach statistical significance in specific age ranges. Mediation analysis verified that TG exerted a complete mediating role in the association pathway between age and UA, which was statistically significant exclusively in females (indirect effect = 4.435, 95% CI: 4.214 to 4.679, *p* < 0.001), while no such mediating effect was observed in males (indirect effect = 0.101, 95% CI: −0.007 to 0.210, *p* > 0.05).

**Conclusion:**

Some CBC and biochemical indicators show meaningful age- and sex-specific variation in mid-to-moderate altitude physical examination populations.

## Introduction

High-altitude hypoxia is a unique environmental factor that affects human physiological functions (1). With long-term residence in high-altitude regions, the human body is persistently exposed to an environment characterized by low atmospheric pressure and the resultant reduced partial pressure of oxygen. To acclimatize to hypoxic conditions, the body triggers a series of adaptive and compensatory physiological response, including erythrocytosis, hemorheological alterations (2), and metabolic pathway remodeling (3). Liver function, renal function, blood lipids, uric acid, and CBC represent the most fundamental screening indicators in routine clinical practice. They not only reflect the functional status of major organs, but also serve as core biomarkers for a spectrum of chronic conditions, including fatty liver disease, hyperuricemia, hyperlipidemia, high-altitude polycythemia, and cardiovascular and cerebrovascular diseases (4, 5). In addition to genetic and lifestyle determinants, high-altitude exposure significantly elevates the risk of cardiovascular and metabolic disorders such as hypertension and diabetes mellitus (6, 7). Although a number of relevant studies on high-altitude populations have been conducted, the current body of evidence has notable limitations. First, many previous studies were conducted in populations at extremely high altitudes (> 3,500 meters), such as the Zang nationality. However, the hypoxic stimulation at moderate to high altitudes is much milder, and current evidence for populations at such altitudes is still limited. Second, prior research has predominantly focused on between-group comparisons of single hematological parameters or isolated biochemical indicators. Multi-indicator studies that systematically integrate liver function, blood lipids, uric acid, and the full CBC panel remain relatively scarce.

Numerous studies have confirmed that age and sex are two core factors of human CBC and biochemical indicators, and this association has been extensively validated in populations across different regions worldwide (8). Aging is a complex process, which is not only influenced by internal genetic factors, but also shaped by external environmental exposure (9). It is characterized by progressive deterioration across all functional domains of biological systems over time, and is accompanied by gradual functional decline of multiple organs (10).

Laboratory test indicators are of critical clinical significance. With advancing age, human hematopoietic function, hepatic and renal functions, as well as anabolic and metabolic processes undergo corresponding alterations (8–11). Meanwhile, the decline in metabolic rate during middle and old age gives rise to stage specific fluctuations in various laboratory indicators (12). With respect to sex, the age-related trajectories of test indicators differ between males and females, driven by disparities in physiological structure, basal metabolic rate, and hormonal profiles. Accumulating evidence indicates that advancing age and sex do not act independently; instead, they exert interactive effects that further diverge the changing patterns of indicators between males and females across different age groups (13). Sexual dimorphism is not confined to phenotypic differences in somatosensory perception and behavioral traits between males and females; it also modulates metabolic regulation across all organ systems (14). Males and females exert selective regulatory effects on lipid metabolism, purine metabolism, and hepatocyte function (15). In reproductive age females, estrogen binds to estrogen receptor α (ERα) in hepatocytes, which inhibits aberrant hepatic lipid synthesis, promotes renal excretion of uric acid, and attenuates lipotoxic injury to hepatocytes, thus exerting comprehensive protective effects on multiple metabolic pathways, this is a key sex-difference mechanism underlying the metabolic protective advantage in females (16, 17). During perimenopause and postmenopause periods, the estrogen levels in women decline. The reduction in estrogen can lead to lipid metabolism disorders, which increase the risk of cardiovascular diseases such as hypertension. Lipid metabolism disorders may be risk factors for elevated serum uric acid levels (18, 19). There is no protective effect of estrogen deficiency mediated by ERα in male bodies. Accordingly, dyslipidemia, hyperuricemia, and hepatocyte injury are likely to form an interconnected regulatory network with mutual influences, and the coordinated variation between lipid metabolism indicators and liver enzymes is far more pronounced in males than in females, highlighting significant sex-based differences (20). Epidemiological studies have revealed that the strength of inter-indicator correlations changes dynamically with age, and that metabolic profiles differ across age groups and between sexes. As people age, their circulating triglyceride levels change, and elevated triglycerides can further increase blood uric acid levels (11, 21). Moreover, during the perimenopausal period in women, the decline in estrogen remodels lipid metabolism and purine metabolism, which may lead to gender-dependent “age—triglyceride—uric acid” pathways. We hypothesize that the age-dependent increase in UA in women is partially mediated by the concurrent increase in TG, reflecting estrogen-related metabolic changes; while in men, the effect of age on UA is more direct and not dependent on TG. Sex plays a pivotal role in male and female metabolism, and represent the core mechanism driving the divergence in indicator distribution and inter-indicator correlation patterns.

Building on the research gaps outlined above, the present study leverages a large-scale medical examination dataset of 83,945 individuals to perform systematic stratified analyses by sex and age, with the objectives of accurately identifying the key inflection points of indicator changes and exploring inter-indicator correlation patterns across different sexes and age groups.

## Methods

### Survey description

This study is a cross-sectional study. This study protocol complies with the guidelines of the Declaration of Helsinki and was approved by the Ethics Committee of the Sixth Affiliated Hospital of Kunming Medical University (China, trial registration number: 2021-kmykdx6f-04). All participants provided written consent after being fully informed.

### Participant enrollment

A total of 83,945 individuals undergoing physical examination in mid-to-high-altitude areas of Yuxi City, southwestern China (1,500–1,800 m above sea level), from 2013 to June 2018 were enrolled, including 32,153 females and 51,792 males. Inclusion criteria were as follows: (1) Residing in mid-to-high-altitude areas; (2) Long-term local residents (with continuous residence in the region for at least 1 year) aged ≥18 years; and (3) Completed full biochemical panel and CBC tests in this examination with complete available data. Exclusion criteria were as follows: (1) Women in the puerperal period; (2) Rheumatic heart disease and systemic lupus erythematosus and other autoimmune diseases; (3) Severe organ failure and systemic infection; and (4) Suffering from mental illness and unable to cooperate with the research-related procedures and complete the questionnaires.

### Laboratory detection

The clinical laboratory has obtained laboratory accreditation from the China National Accreditation Service for Conformity Assessment (CNAS), and its whole-process quality management system complies with national clinical laboratory accreditation standards. During the study period, the laboratory continuously participated in external quality assessment (EQA) programs organized by the National Center for Clinical Laboratories of the National Health Commission and the Yunnan Provincial Center for Clinical Laboratories. All EQA results for all tested analytes were qualified across all study years. Daily internal quality control (IQC) was performed on all testing analyzers. The instrument models remained stable throughout the study with no major equipment replacement. The CNAS Laboratory Accreditation Certificate is available upon request to the corresponding author.

### Clinical features

The data collected in this study include socio-demographic information such as sex and age. The biochemical, lipid, metabolic indicators measured using the Roche cobas 8,000 automated biochemical analyzer (Roche Diagnostics, Mannheim, Germany). The tested biochemical indicators fell into the following categories: liver function parameters: total bilirubin (TBIL), direct bilirubin (DBIL), indirect bilirubin (IBIL), total protein (TP), albumin (ALB), globulin (GLB), alanine aminotransferase (ALT), aspartate aminotransferase (AST), alanine aminotransferase/aspartate aminotransferase ratio (ALT/AST), aspartate aminotransferase/alanine aminotransferase ratio (AST/ALT), alkaline phosphatase (ALP), γ-glutamyl transpeptidase (GGT), and α-L-fucosidase (AFU); Renal function parameters: urea (Urea), creatinine (CREA), and uric acid (UA); Lipid profile parameters: total cholesterol (TC), triglyceride (TG), high-density lipoprotein cholesterol (HDL), low-density lipoprotein cholesterol (LDL), very low-density lipoprotein cholesterol (VLDL), apolipoprotein A1 (ApoA1), apolipoprotein B100 (ApoB100), and lipoprotein(a) [Lp(a)]; Blood glucose parameter: glucose (GLU). Hematological parameters were measured using the Mindray BC-6800 automatic hematology analyzer (Mindray Medical International, Shenzhen, China), including white blood cell count (WBC), neutrophil percentage (NEUT), lymphocyte percentage (LYM), monocyte percentage (MONO), eosinophil percentage (EO), red blood cell count (RBC), hemoglobin (Hb), hematocrit (HCT), mean corpuscular volume (MCV), mean corpuscular hemoglobin (MCH), mean corpuscular hemoglobin concentration (MCHC), platelet count (PLT), red cell distribution (RDW), platelet distribution width (PDW), mean platelet volume (MPV), plateletcrit (PCT), Platelet–large cell ratio (P–LCR).

### Grouping

To analyze the age and sex dependent variations in CBC and biochemical indicators, participants of each sex were stratified into seven age groups at 10-year intervals: 18–25 years, 26–35 years, 36–45 years, 46–55 years, 56–65 years, 66–75 years, and >75 years.

### Statistical analysis

Statistical Product and Service Solutions (version: 29.0) and R language (version: 4.6.0) were used for all analyses. For categorical data, between-group comparisons were performed using the chi-square test. For normally distributed continuous data, the independent samples *t*-test was used for two-group comparisons, and one-way ANOVA was applied for multi-group comparisons. For skewed measurement data, two-group comparisons were conducted using the Mann–Whitney U test, while multi-group comparisons were performed using the Kruskal–Wallis H test. Two-way nonparametric tests were employed to analyze the main effects of age and sex, as well as their interaction effect, on each laboratory indicator. When the overall multi-group comparison reached statistical significance, Dunn’s *post hoc* test was further performed for pairwise comparisons between groups, with Bonferroni correction applied for multiple testing. Bonferroni correction was also adopted to adjust for repeated male–female comparisons within each age group. An adjusted *p* value <0.05 was set as the threshold for statistically significant differences. Age trend inflection point plots were constructed with age groups on the x-axis and the median value of each indicator on the y-axis, to characterize the changing trajectories and identify key age inflection points. Stratified by sex and age group, Spearman rank correlation analysis was applied to examine inter-correlations among indicators. Indicator pairs with a correlation coefficient *r* > 0.3 and *p* < 0.05 were defined as moderate correlations with clinical significance, and correlation heat maps were generated based on this criterion. Stratified by sex, the Bootstrap method with 5,000 resamples was employed to explore the sex heterogeneity of the mediating effect of TG on the association between age and UA. The follow-up study design is shown in Figure 1.

**Figure 1:**
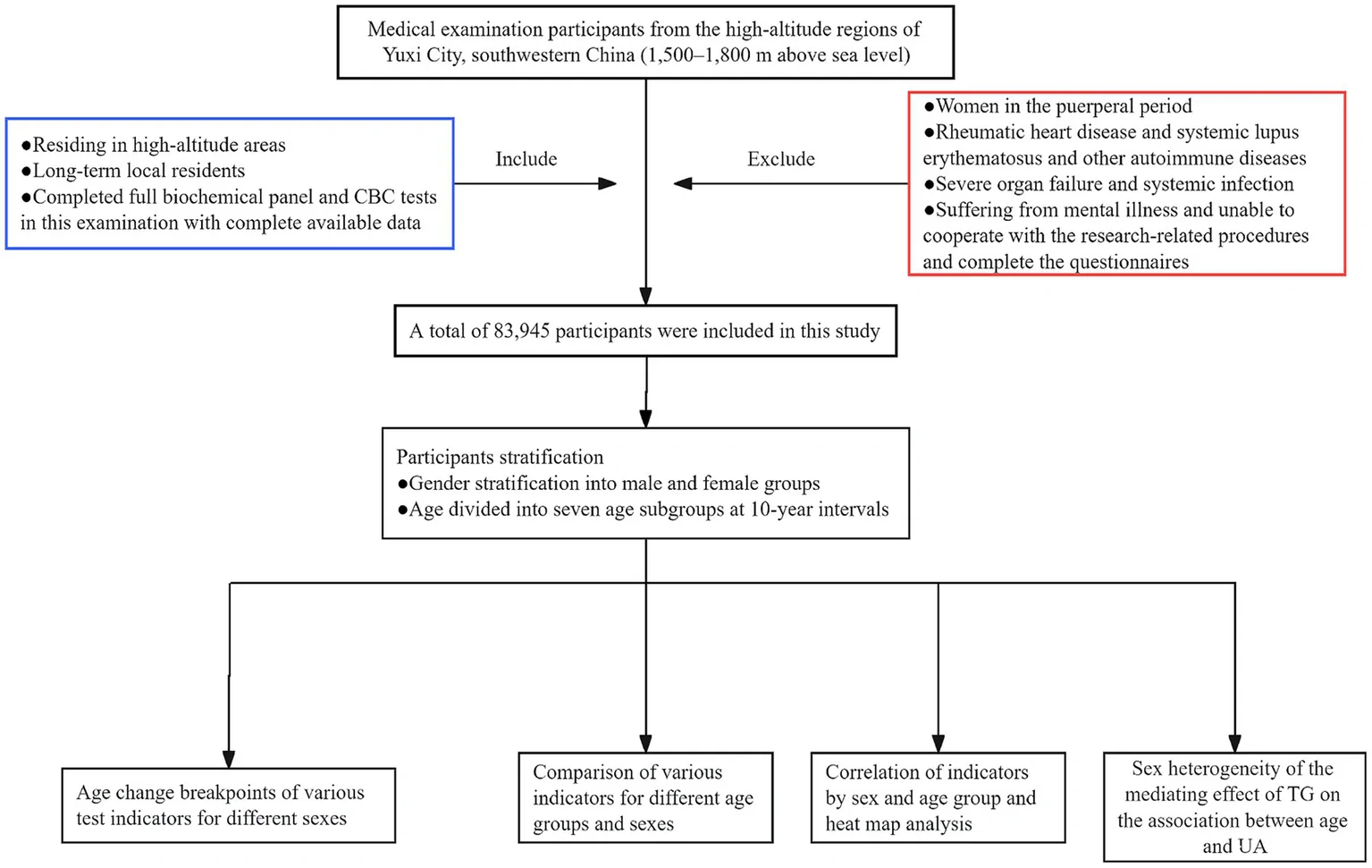
Flow chart of the study design. CBC, complete blood count.

## Results

### Comparison of various indicators for different age groups and sexes

Comparisons of CBC and biochemical indicators between males and females across age groups in the physical examination mid-to-high-altitude population are presented in Supplementary Table S1. The patterns of sex differences in CBC parameters and biochemical indices differ slightly across age groups. All between-group comparisons were corrected using the Bonferroni method, with adjusted *p* < 0.05 considered statistically significant.

To distinguish statistical significance from clinical relevance, we calculated the relative median difference (RMD) between males and females. A sex difference was considered clinically meaningful only when adjusted *p* < 0.05 and |RMD| > 10%. The results showed that although most indicators exhibited statistically significant sex differences across multiple age groups (adjusted *p* < 0.05) in Supplementary Table S2.

In the 18–25 years, all included renal function and lipid profile indices showed statistically significant sex differences between males and females. For liver function indices, significant differences were observed in all parameters except TP; for CBC parameters, significant differences were found in all indices except WBC, NEUT, LYMPH, EO, MCH, and MCHC. No statistically significant sex difference was detected in GLU. In this age group, indicators with |RMD| > 10% included TBIL, DBIL, IBIL, ALT, AST, ALT/AST, AST/ALT, ALP, Urea, CREA, UA, TG, HDL, LDL, VLDL, APOB100, and Lp(a).

In the 26–35 years, statistically significant sex differences were identified in all included renal function and lipid profile indices. For liver function indices, significant differences were present in all parameters except TP; for CBC parameters, significant differences were observed in all indices except WBC, NEUT, LYMPH, EO, MCV, MCH, MCHC, and PCT. No statistically significant sex difference was found in GLU. In this age group, indicators with |RMD| > 10% included TBIL, DBIL, IBIL, ALT, AST, ALT/AST, AST/ALT, ALP, GGT, AFU, Urea, CREA, UA, TG, HDL, LDL, VLDL, APOB100, and Lp(a).

In the 36–45 years, all included renal function, liver function, and lipid profile indices, as well as GLU, showed statistically significant sex differences. For CBC parameters, significant differences were detected in all indices except WBC, NEUT, LYMPH, EO, MCV, MCH, MCHC, PLT, and PCT. In this age group, indicators with |RMD| > 10% included TBIL, DBIL, IBIL, ALT, AST, ALT/AST, AST/ALT, ALP, GGT, AFU, Urea, CREA, UA, TG, HDL, LDL, VLDL, and APOB100.

In the 46–55 years, all included renal function indices, lipid profile indices, and GLU exhibited statistically significant sex differences. For liver function indices, significant differences were found in all parameters except TC; for CBC parameters, significant differences were present in all indices except WBC, NEUT, LYMPH, EO, MCHC, PLT, MPV, PDW, PCT and P-LCR. In this age group, indicators with |RMD| > 10% included TBIL, DBIL, IBIL, ALT, ALT/AST, AST/ALT, CREA, UA, TG, HDL, and VLDL.

In the 56–65 years, all included renal function indices showed statistically significant sex differences. For liver function indices, significant differences were observed in all parameters except AST; for lipid profile indices, significant differences were present in all parameters except TG, APOB100 and VLDL. For CBC parameters, significant differences were found in all indices except WBC, NEUT, LYMPH, EO, MONO, RBC, HB, HCT, MCV, MCH, MCHC, RDW, PLT, MPV, PCT, PDW, and P-LCR. No statistically significant sex difference was detected in GLU. In this age group, indicators with |RMD| > 10% included TBIL, DBIL, ALT, ALT/AST, CREA, UA, and HDL.

In the 66–75 years, all included renal function indices, lipid profile indices exhibited statistically significant sex differences. For liver function indices, significant differences were found in all parameters except TP, ALB, AFU; for lipid profile indices, significant differences were present in all parameters except Lp(a). For CBC parameters, significant differences were present in all indices except WBC, NEUT, LYMPH, EO, MONO, RBC, HB, HCT, MCV, MCH, MCHC, RDW, PLT, MPV, PCT, PDW, and P-LCR. In this age group, indicators with |RMD| > 10% included TBIL, DBIL, ALT, ALT/AST, CREA, UA, HDL, and Lp(a).

In the >75 years, all included renal function indices showed statistically significant sex differences. For liver function indices, significant differences were present in all parameters except TP, GLB, ALB, ALT, AST, ALT/AST, ALP and AFU. For lipid profile indices, significant differences were found in all parameters except LDL, APOB100, and Lp(a). For CBC parameters, significant differences were observed in all indices except WBC, NEUT, LYMPH, EO, MONO, RBC, HB, HCT, MCV, MCH, MCHC, RDW, PLT, MPV, PCT, PDW, and P-LCR. No statistically significant sex difference was detected in GLU. In this age group, indicators with |RMD| > 10% included TBIL, DBIL, ALT, CREA, UA, TG, and VLDL.

Pairwise comparisons across age groups within the same sex showed that most indicators changed significantly with age. Taking TBIL as an example. In males, Significant differences were observed between the 46–55 and 36–45 years groups, between the 56–65 and 46–55 years groups, between the 66–75 and 56–65 years groups, and between the >75 and 66–75 years groups after Bonferroni correction (*p* < 0.05). In females, significant differences were found between the 56–65 and 18–25 years groups and between the 66–75 and 26–35 years groups after Bonferroni correction (*p* < 0.05). The results of pairwise comparisons of other indicators among different age groups are presented in Supplementary Table S3.

### Age change breakpoints of various test indicators for different sexes

The trends of various indicators of CBC and biochemical tests in physical examination individuals in mid-to-high-altitude areas with respect to age are shown in Figure 2. For the vast majority of CBC and biochemical indicators, age-related trajectories were largely consistent between males and females, and fell into five main patterns: continuous increase, continuous decrease, fluctuation across all age strata, increase followed by decrease, and decrease followed by increase. The age inflection points were also generally concordant between the two sexes. In CBC parameters, RBC, HB, and HCT show clear gender differences. In the age groups of 18–25 to 56–65 years, male levels are consistently higher than those of females, while a reversal occurs in the 66–75 age group, with females surpassing males. In biochemical indicators. However, in biochemical indicators ALP, GLB, TG, and UA Show significant gender differentiation in their age dependent evolution patterns. ALP: Levels increased steadily in males, whereas they declined in females after the 56–65 years. GLB: Levels exhibited a steady upward trend in males, but decreased in females after the 66–75 years. TG: In females, levels rose steadily followed by a slight decline after the 66–75 years; in males, the inflection point marking the transition from increase to decrease occurred at the 46–55 years. UA: Overall, levels in males showed a general declining trend with a slight rebound after the 56–65 years; in females, the trend reversed from increase to decrease after the 66–75 years.

**Figure 2:**
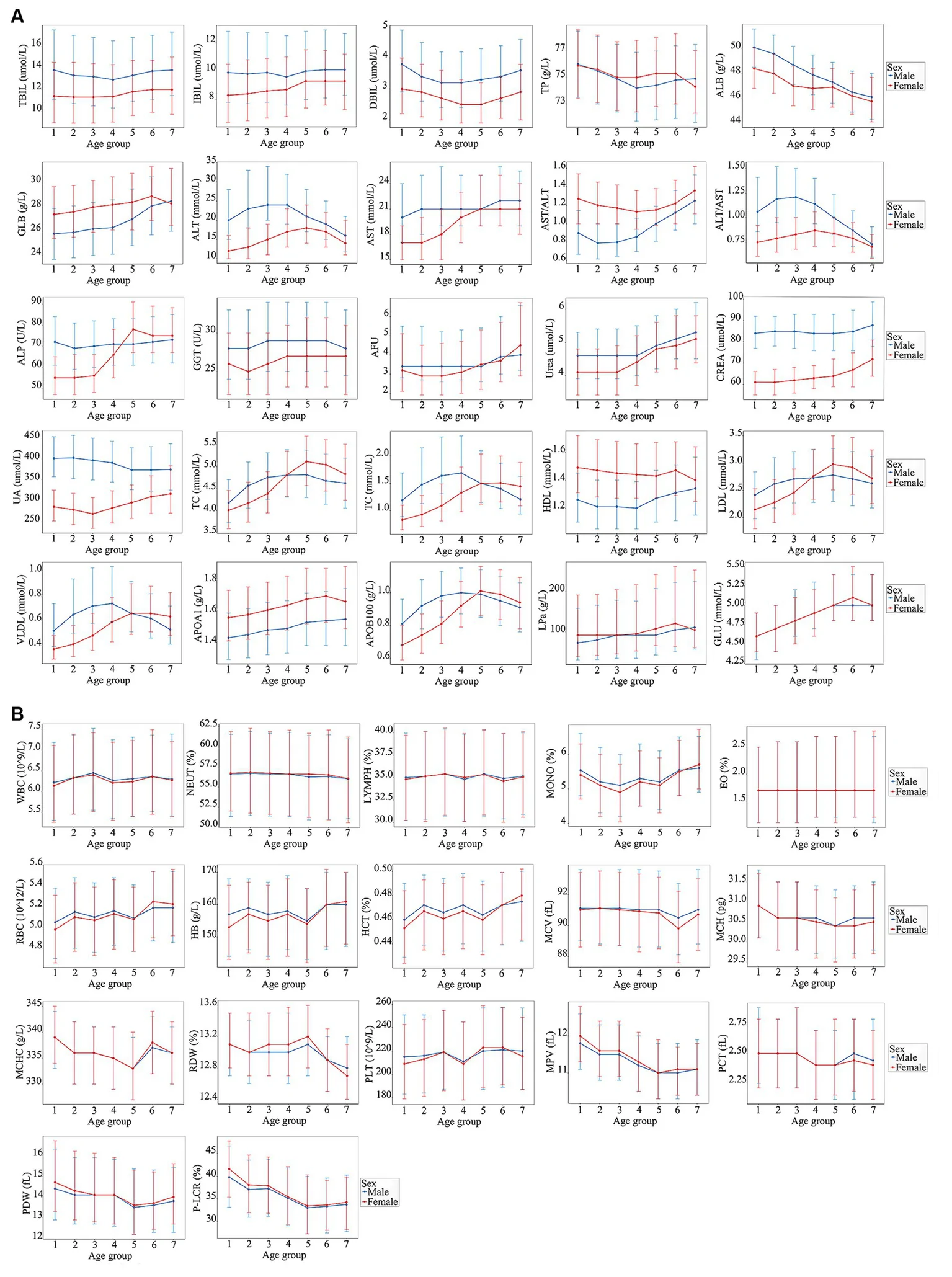
Broken-line plot of median values for laboratory parameters across age groups stratified by sex. The x-axis represents 7 age categories: 1 (18–25 years), 2 (26–35 years), 3 (36–45 years), 4 (46–55 years), 5 (56–65 years), 6 (66–75 years), and 7 (>75 years). The y-axis denotes complete blood count (CBC) and biochemical parameters. Males are marked in blue, and females in red. **(A)** Break-point plot of biochemical indicators. **(B)** Break-point plot of complete blood count.

### Correlation of indicators by sex and age group and heat map analysis

To further explore the interrelationships among CBC and biochemical indicators, we conducted correlation analyses stratified by sex and age group. For indicator pairs across liver function, renal function, lipid profile, blood glucose and CBC panels with a correlation coefficient *r* > 0.3 and statistical significance, correlation heat maps (Figure 3) were generated to visualize the age-dependent trends of inter-indicator association strength. Bonferroni correction was applied for multiple comparisons among these indicators, with a corrected *p* < 0.05 set as the threshold for statistical significance in Supplementary Tables S3, S4. Correlation analyses revealed prominent sex differences in the co-variation patterns of metabolic indicators. In females, only a transient moderate positive correlation between UA and TG or VLDL was detected during the perimenopausal period (36–45 years, *r* = 0.31), with weak associations observed in other age strata. The negative correlation between DBIL and ApoB100 reached a moderate magnitude exclusively in the advanced age group (>75 years, *r* = −0.31), and showed no evident association with perimenopausal hormonal fluctuations (Figure 2A). After Bonferroni correction, all variable pairs with |*r*| > 0.3 remained statistically significant across all age groups in females (corrected *p* < 0.05). In contrast, the correlations between lipid metabolism indicators (TG, VLDL, ApoB100) and liver enzymes (ALT, AST/ALT ratio, ALT/AST ratio) were strongest in young males aged <25 years, with *r* values generally ranging from 0.31 to 0.39; these correlations gradually attenuated with advancing age. The associations between UA and TG or VLDL persisted across all age strata in males, and remained at a moderate level even in the older adults population (*r* = 0.30) (Figure 3B). In males, the correlation between HDL and AST/ALT was no longer statistically significant in the >75-year age group after correction; similarly, the correlations of ApoB100 with AST/ALT, ApoB100 with ALT/AST, and ApoB100 with ALT did not reach statistical significance in either the 66–75-year or >75-year age groups (corrected *p* > 0.05).

**Figure 3:**
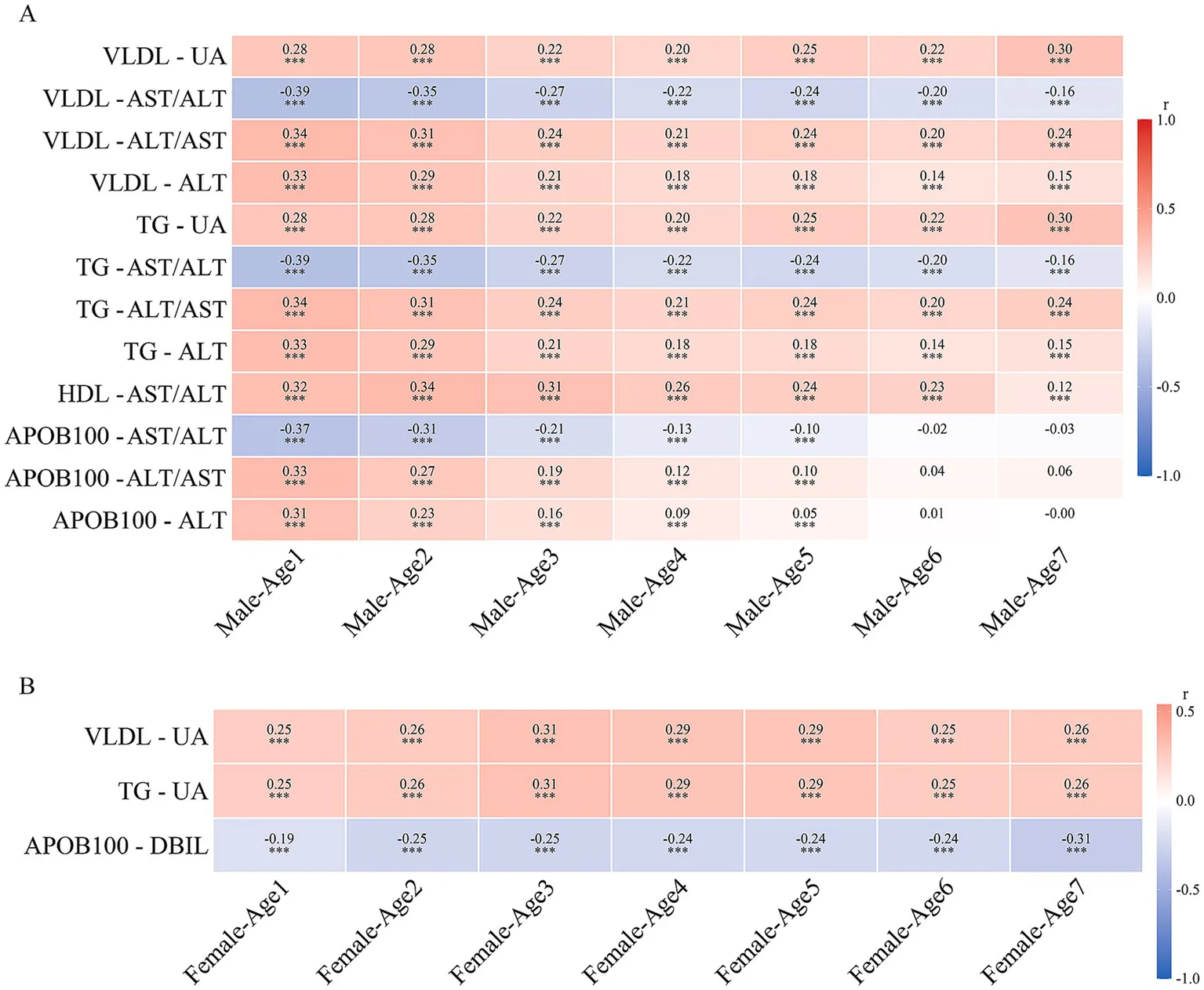
Correlation heat maps of parameters across different age groups in males and females (r > 0.3, *p* < 0.05). Orange indicates positive correlations, while blue indicates negative correlations. Color intensity corresponds to the strength of association. **(A)** Correlation heat map in males. **(B)** Correlation heat map in Females.

### Sex heterogeneity of the mediating effect of TG on the association between age and UA

Distinct sex-specific inflection points and trend patterns were observed for TG and UA, along with a close correlation between the two indicators. The Bootstrap method (5,000 resampling iterations) was applied to test the mediating effect. A mediation model was constructed with TG as the mediating variable to explore the mechanism underlying the effect of age on UA. The results are presented in Table 1. Distinct patterns emerged between the two sex groups. The sex-stratified mediation pathways are illustrated in Figure 4.

**Table 1:** Results of the parallel mediation analysis.

Bootstrap	Age→ TG → UA				
	*β*	SE	95% CI	*P*	Conclusion
Male					
Total effect	−6.8826	0.2611	(−7.388, −6.370)	<0.05	No mediation
Direct effect	−6.984	0.255	(−7.469, −6.488)	<0.05	
Indirect effect	0.101	0.056	(−0.007, 0.210)	>0.05	
Female					
Total effect	4.524	0.292	(3.944, 5.086)	<0.05	Complete mediation
Direct effect	0.088	0.301	(−0.509, 0.680)	>0.05	
Indirect effect	4.435	0.118	(4.214, 4.679)	<0.05	

Age; TG, Triglyceride; UA, Uric acid; B, estimate of the regression coefficient; SE, standard error of estimate; CI, confidence interval.

**Figure 4:**
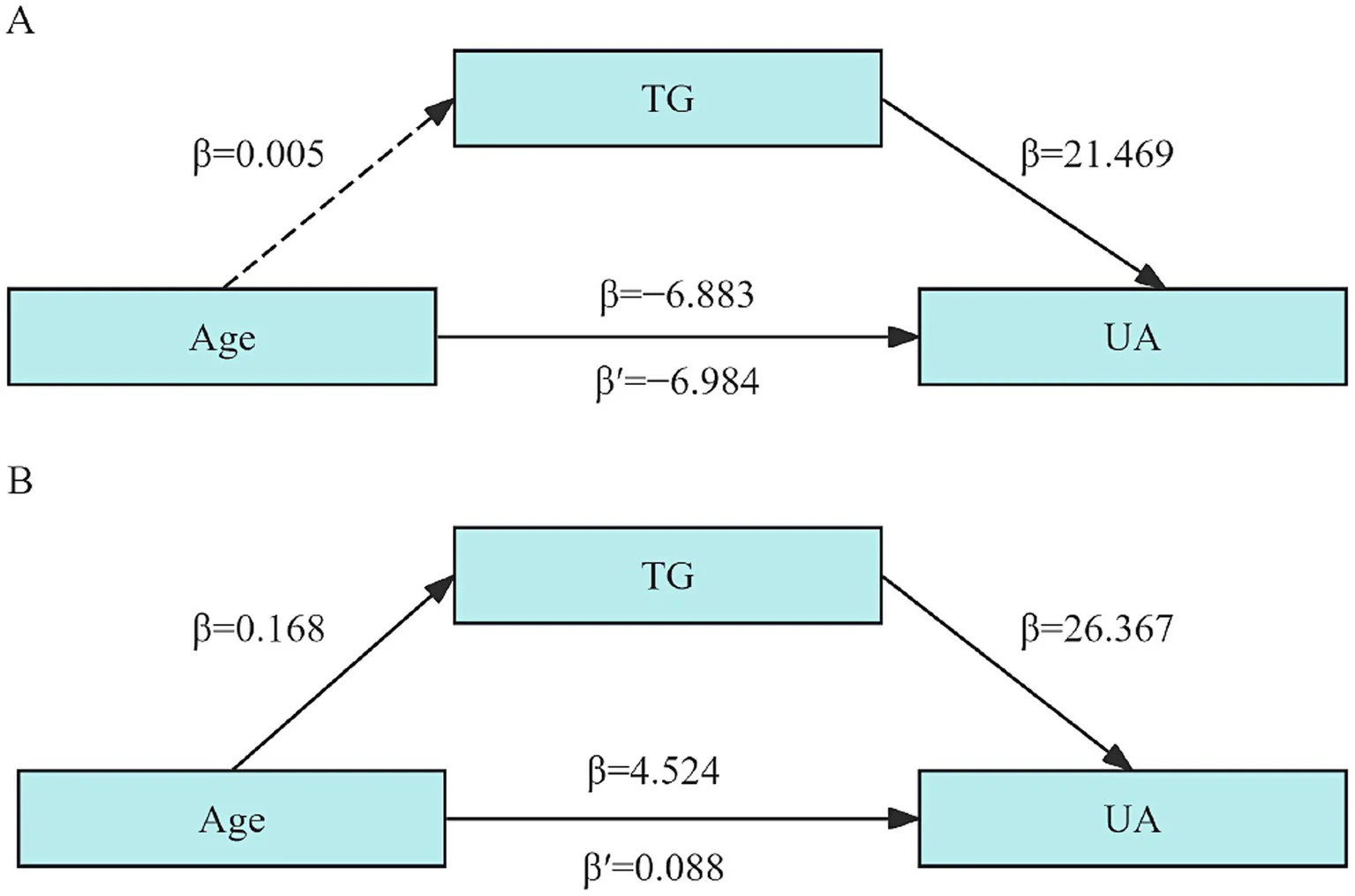
Comparison of the mediation pathways between age, TG, and UA by gender. **(A)** Model in male. **(B)** Model in female. Solid arrows indicate significant paths (*p* < 0.05); dotted arrows indicate nonsignificant paths.

In males, the total effect of age on UA was statistically significant (c = −6.883, *p* < 0.001), indicating that age exerts a significant influence on UA levels. The effect of age on TG did not reach statistical significance (path a: β = 0.005, *p* > 0.05), whereas the effect of TG on UA was statistically significant (path b: β = 21.469, *p* < 0.001). The indirect effect was not statistically significant (ind_eff = 0.101, 95% CI: −0.007 to 0.210, *p* > 0.05), while the direct effect remained significant (c′: β = −6.984, 95% CI: −7.469, −6.488, *p* < 0.001). These findings indicate that the effect of age on UA is not mediated through TG; that is, TG does not exert a mediating effect between age and UA in males, and the association is entirely driven by the direct effect of age on UA.

In females, the total effect of age on UA was statistically significant (c = 4.524, *p* < 0.001), confirming a significant influence of age on UA levels. The indirect effect was statistically significant (ind_eff = 4.435, 95% CI: 4.214, 4.679, *p* < 0.001). Both the effect of age on TG (path a: β = 0.168, *p* < 0.001) and the effect of TG on UA (path b: β = 26.367, *p* < 0.001) were statistically significant. After incorporating the mediating variable TG, the direct effect became non-significant (c′ = 0.088, 95% CI: −0.509, 0.680, *p* > 0.05). These results demonstrate that the effect of age on UA is mediated through TG, indicating that TG plays a full mediating role in the relationship between age and UA among females. However, due to the influence of the cross-sectional study, what it reflects is the correlation pattern rather than the causal relationship.

## Discussion

High-altitude triggers a spectrum of acute and chronic compensatory responses in the human body to sustain survival, which exerts multifaceted effects on human physiology and may contribute to the onset of various disorders (22). CBC and biochemical reference intervals are of critical value for clinicians and researchers to interpret laboratory results and monitor therapeutic efficacy, enabling early detection of a wide range of systemic diseases. Accumulating population based evidence has demonstrated marked geographic heterogeneity in the baseline distribution of laboratory parameters. Reference intervals derived from populations in other geographic settings cannot be directly generalized across regions; therefore, establishing CBC indicators for populations living at high altitudes is of great significance for the prevention and management of high-altitude diseases (23, 24). Under high-altitude conditions, hypoxia stimulates a cascade of compensatory reactions in the human hematologic system, and hematologic alterations represent a core adaptive change. Meanwhile, most biochemical parameters undergo significant changes after acute high-altitude exposure, which also play an indispensable role in high-altitude acclimatization (25, 26). Taken together, establishing age and sex stratified CBC and biochemical reference intervals for physical examination high-altitude populations can improve the diagnostic accuracy of disease screening, chronic mountain sickness and metabolism-related chronic diseases among plateau residents. This work carries important implications for the regional comprehensive prevention and control of high-altitude diseases as well as individualized health management.

Numerous studies focusing on CBC and biochemical indicators in high-altitude populations have been published. Yuan et al. conducted a 3 year retrospective analysis of changes in CBC indices, liver and kidney function, blood lipids, myocardial enzyme profiles, and rheumatoid factor levels among 114 high-altitude Tibetans and 93 high-altitude Han Chinese residents. The study revealed significant differences in hematological and biochemical profiles between Tibetans and Han individuals with long-term residence in extremely high-altitude areas (27). Nipa Basak et al. performed correlation analysis of CBC parameters in 168 participants from high-altitude regions and 89 from low-altitude regions, to investigate hematological differences between Tibetan populations at high and low altitudes. The results showed significant disparities in RBC, HCT, Hb, and MCHC in both sexes, as well as in MCV in females. Specifically, RBC, HCT, and Hb concentrations were significantly higher, while MCV was significantly lower in both males and females at high altitude compared with their low-altitude counterparts (28). Wan et al. investigated the effects of altitude and duration of residence on CBC indices among acclimatized individuals living at different altitudes on the plateau. The findings demonstrated that hematological parameters changed dynamically with altitude, and the length of residence influenced the stabilization of hematological indices after migration (29). Meng et al. explored factors associated with hyperuricemia based on 284 high-altitude residents. The results indicated that sex, age, red blood cell count, and creatinine were independently associated with high-altitude hyperuricemia (30).

Compared with previous studies, this research has achieved significant improvements and innovations in terms of the research subjects and methods, as follows: (1) The sample size of this research is large; (2) The included blood routine and biochemical indicators are comprehensive and systematic, not just single inclusion of blood routine or biochemical indicators; (3) Gender and age were stratified, and detailed stratification was carried out, and the turning points of age conversion for each indicator were quantitatively identified through the breakpoints chart; and (4) This study fills the research gap regarding age- and sex-related differences in laboratory parameters among permanent Han Chinese residents receiving routine medical examinations in mid-to-high-altitude areas of southwestern China. Notably, most previous plateau-related studies were conducted in high-altitude populations (>3,500 m, e.g., Tibetan populations), where hypoxic stress is substantially stronger than in our moderate-altitude cohort (1,500–1,800 m); therefore, the direct comparability between the two is limited. Meanwhile, multi-indicator studies that systematically integrate liver function, blood lipids, uric acid, the complete CBC panel remain relatively scarce in moderate-altitude regions, and the present study also fills this evidence gap.

The present study found that the age-related trends and breakpoints of most indicators were generally consistent between males and females, characterized by persistent increase or decrease, fluctuation, or phased increase and decrease. This indicates that the effects of aging and high-altitude hypoxia on basic human physiology are universal across sexes. Only four indicators, namely ALP, GLB, TG, and UA, showed unique gender turning points and distinct trend changes, suggesting that sex may serve as key regulators of hepatic lipid metabolism (31, 32), bone metabolic activity (33), and purine metabolism (19), accounting for the sex based differences in parameter distributions. In males, the correlations between lipid metabolism such as TG, VLDL, APOB100, HDL and liver enzymes as well as liver enzyme ratios were strong during adolescence and declined with advancing age. The correlations between male VLDL and TG and UA were the strongest at over 75 years, while in females, the correlation was strongest between 36 and 45 years. APOB100 was naturally positively correlated with DBIL, and this correlation was the strongest at over 75 years, indicating that this correlation is independent of postmenopausal estrogen levels. These differences reflect the selective regulatory effect of estrogen on metabolic pathways: males lack estrogen mediated protection, while estrogen binds to estrogen receptor α (ERα) in hepatocytes and alleviates hepatocellular lipotoxicity by inhibiting lipogenesis and promoting β-oxidation (34). Triglycerides are positively associated with hyperuricemia in both sexes, and this association is age dependent, particularly in females (11). Blood lipids and bilirubin present an inherent negative correlation, as bilirubin can reduce circulating lipid levels by promoting hepatic fat utilization (35). Under hypoxic conditions, the human body can enhance blood oxygen-carrying capacity by increasing red-blood-cell counts per unit blood volume, so as to guarantee tissue oxygen supply. In our mid-to-moderate-altitude study population, baseline RBC and Hb levels were elevated compared with those observed in plain-dwelling populations, and values were consistently higher in males than in females among the young and middle-aged individuals in all age groups. Such sex-related differences in erythrocyte parameters are closely associated with androgen-stimulated erythropoiesis in males and estrogen-mediated regulation of hematopoiesis in females (36, 37). The mediating relationship among age, TG, and UA reveals distinct mechanisms between males and females. In males, age exerts a direct effect on UA independent of TG mediation. In females, by contrast, age influences UA levels through the mediating role of TG, indicating a significant indirect effect.

We recognize several limitations in this study. First, this study adopted a cross-sectional design, which only captures the status of laboratory parameters at a single time point and precludes the inference of causal relationships underlying parameter changes. Second, this study did not collect confounding factors such as smoking, drinking, and BMI, so they were not included in the statistical model for correction. This led to a reduction in the correlation between age and the indicators. Third, only routine liver and kidney function, blood lipid profiles, and CBC were included for phenotypic analysis, while molecular indicators such as hormone levels, inflammatory cytokines, and genetic biomarkers were not examined. Fourth, the inclusion criteria for this study were merely “continuous residence for ≥1 year”, and no information was collected regarding the ethnic composition of the participants, their indigenous/migrant status, or the exact duration of continuous residence. This may lead to confounding effects among different groups at different adaptation stages, thereby affecting the interpretability of the results. Fifth, the mediation analysis in this study is based on a cross-sectional design rather than a confirmation of causal relationships. Accordingly, this study can only characterize population distributions and correlation patterns at the macroscopic level, and cannot elucidate the intrinsic pathways through which sex hormones and hypoxia modulate sex differences in relevant parameters at the molecular level. For future research directions, first, measurements of sex hormone levels and inflammatory cytokines can be incorporated, and confounding factors including smoking status, alcohol consumption, BMI, and history of chronic diseases can be included for multivariable adjustment, to explain sex-based differences in hepatobiliary metabolism, immune status, lipid metabolism, and purine metabolism. Second, longitudinal study can be performed to dynamically observe the evolutionary trends and transition mechanisms of various parameters across different sexes and age groups, clarify the metabolic outcome characteristics at critical age nodes, and further validate the stability of the age breakpoints identified in this study. Finally, building on the parameter correlation patterns established in this study and the synergistic variations of multiple interrelated indicators, early screening models for metabolic abnormalities and altitude-related adaptive alterations tailored to mid-to-high-altitude populations can be constructed. These models will provide a baseline reference for age- and gender-based health management and early risk stratification of chronic diseases within the region.

## Conclusion

In summary, the biochemical and blood routine indicators of physical examination individuals in early screening models for metabolic abnormalities and high-altitude adaptive injury tailored to mid-to-high-altitude populations can be constructed.-altitude areas show significant age dependent and gender specific characteristics. However, as we did not adjust for confounding factors including BMI, lifestyle factors, and chronic disease history, these associations should not be considered independent effects. Sex may be the cause of the specificity. The mediation analysis indicates that TG may play a mediating role in the association between female age and UA, it is impossible to establish a causal relationship. Future research can include multi-altitude gradient and multi-center studies as well as longitudinal studies are still needed in the future to better interpret this conclusion.

## Data Availability

Data availability is restricted due to patient privacy regulations. Relevant information can be obtained from the corresponding author upon reasonable request. Requests to access these datasets should be directed to YK, kuangye@kmmu.edu.cn.

## Glossary

CBC: Complete blood count
TBIL: Total bilirubin
DBIL: Direct bilirubin
IBIL: Indirect bilirubin
TP: Total protein
ALB: Albumin
GLB: Globulin
ALT: Alanine aminotransferase
AST: Aspartate aminotransferase
ALT/AST: ALT/AST ratio
AST/ALT: AST/ALT ratio
ALP: Alkaline phosphatase
GGT: γ-glutamyl transpeptidase
AFU: α-L-fucosidase
Urea: Urea
CREA: Creatinine
UA: Uric acid
TC: Total cholesterol
TG: Triglyceride
HDL: High-density lipoprotein cholesterol
LDL: Low-density lipoprotein cholesterol
VLDL: Very low-density lipoprotein cholesterol
ApoA1: Apolipoprotein A1
ApoB100: Apolipoprotein B100
Lp(a): Lipoprotein(a)
GLU: Glucose
WBC: White blood cell count
NEUT: Neutrophil percentage
LYM: Lymphocyte percentage
MONO: Monocyte percentage
EO: Eosinophil percentage
RBC: Red blood cell count
Hb: Hemoglobin
HCT: Hematocrit
MCV: Mean corpuscular volume
MCH: Mean corpuscular hemoglobin
MCHC: Mean corpuscular hemoglobin concentration
PLT: Platelet count
RDW: Red cell distribution width
PDW: Platelet distribution width
MPV: Mean platelet volume
PCT: Plateletcrit
P-LCR: Platelet–large cell ratio
