# Supplementary material for: A cross-sectional study of routine hematological and biochemical parameters with age- and sex-specific analyses in mid-to-high-altitude regions of southwestern China
Source: Front Public Health. 2026 Oct 28;14:1949654. doi: 10.3389/fpubh.2026.1949654 (PMC13752126; doi:10.3389/fpubh.2026.1949654)
Supplement: Supplementary file 1 [file Table_1.DOCX]

Table S1: Comparison of various indicators for different age groups and sexes.

| Variables | Sex | Overall | Different age groups | | | | | | |  |  |
| --- | --- | --- | --- | --- | --- | --- | --- | --- | --- | --- | --- |
|  |  |  | 18-25 (years) | 26-35 (years) | 36-45 (years) | 46-55 (years) | 56-65 (years) | 66-75 (years) | >75 (years) | Statistic | P |
| TBIL, umol/L | Male | 13.20 (10.60, 16.80) | 13.80 (11.10, 17.50)a | 13.30 (10.70, 17.00)a* | 13.20 (10.50, 16.80)a* | 12.90 (10.30, 16.50)a*#△ | 13.30 (10.90, 16.80)a*▲ | 13.70 (11.10, 17.00)a△▲ | 13.80 (11.45, 17.30)a#△▲ | 124.8 | <0.001 |
|  | Female | 11.40 (9.00, 14.40) | 11.40 (8.93, 14.50) | 11.30 (8.90, 14.50) | 11.30 (8.90, 14.40) | 11.35 (9.00, 14.30) | 11.80 (9.60, 14.70)*#△▲ | 12.00 (9.90, 14.70)*#△▲ | 12.00 (9.70, 15.00) | 64.95 | <0.001 |
| DBIL, umol/L | Male | 3.30 (2.50, 4.30) | 3.80 (2.90, 4.90)a | 3.40 (2.60, 4.50)a* | 3.20 (2.40, 4.20)a*# | 3.20 (2.40, 4.20)a*# | 3.30 (2.50, 4.30)a*#△▲ | 3.40 (2.60, 4.40)a*△▲☆ | 3.60 (2.90, 4.60)a#△▲☆★ | 513 | <0.001 |
|  | Female | 2.70 (2.00, 3.60) | 3.00 (2.20, 4.00) | 2.90 (2.10, 3.80)* | 2.70 (2.00, 3.60)*# | 2.50 (1.90, 3.30)*#△ | 2.50 (1.90, 3.20)*#△ | 2.70 (2.05, 3.40)*#▲ | 2.90 (2.00, 3.80)▲☆ | 476.44 | <0.001 |
| IBIL, umol/L | Male | 9.80 (7.80, 12.70) | 9.90 (7.80, 12.80)a | 9.80 (7.70, 12.70)a | 9.90 (7.80, 12.70)a | 9.60 (7.60, 12.50)a△ | 10.00 (8.00, 12.80)a#▲ | 10.10 (8.10, 12.90)a#▲ | 10.10 (8.30, 12.65)a▲ | 62.59 | <0.001 |
|  | Female | 8.60 (6.70, 10.90) | 8.30 (6.40, 10.50) | 8.40 (6.50, 10.80) | 8.60 (6.70, 10.90)*# | 8.70 (6.80, 11.00)*# | 9.30 (7.40, 11.50)*#△▲ | 9.30 (7.60, 11.45)*#△▲ | 9.30 (7.28, 11.20)*#△ | 190.15 | <0.001 |
| TP, g/L | Male | 74.90 (72.30, 77.50) | 76.00 (73.50, 78.50) | 75.50 (73.00, 78.10)* | 74.90 (72.40, 77.50)*# | 74.20 (71.70, 76.90)a*#△ | 74.40 (71.80, 77.00)a*#△ | 74.80 (71.90, 77.40)*#▲ | 74.90 (71.60, 77.50)*#▲ | 972.69 | <0.001 |
|  | Female | 75.30 (72.70, 77.90) | 75.90 (73.40, 78.60) | 75.60 (73.10, 78.20) | 75.00 (72.50, 77.70)*# | 75.00 (72.40, 77.80)*# | 75.30 (72.80, 78.00)*△ | 75.30 (73.00, 78.30) | 74.30 (72.30, 77.03)*#☆★ | 196.18 | <0.001 |
| ALB, g/L | Male | 48.50 (46.80, 50.20) | 50.10 (48.50, 51.60)a | 49.60 (48.00, 51.10)a* | 48.70 (47.10, 50.20)a*# | 47.90 (46.30, 49.50)a*#△ | 47.30 (45.60, 48.90)a*#△▲ | 46.50 (44.90, 48.20)*#△▲☆ | 46.10 (44.30, 48.00)*#△▲☆★ | 6764.83 | <0.001 |
|  | Female | 47.30 (45.60, 48.90) | 48.40 (46.80, 50.10) | 48.00 (46.40, 49.60)* | 47.00 (45.40, 48.60)*# | 46.80 (45.10, 48.40)*#△ | 46.90 (45.30, 48.40)*# | 46.20 (44.70, 48.00)*#△▲☆ | 45.75 (44.10, 47.70)*#△▲☆ | 1819.67 | <0.001 |
| GLB, g/L | Male | 26.30 (24.10, 28.60) | 25.80 (23.68, 27.90)a | 25.90 (23.80, 28.10)a | 26.20 (24.00, 28.40)a*# | 26.30 (24.10, 28.60)a*#△ | 27.00 (24.80, 29.50)a*#△▲ | 28.10 (25.70, 30.50)a*#△▲☆ | 28.50 (26.00, 31.20)*#△▲☆ | 1337.67 | <0.001 |
|  | Female | 27.90 (25.80, 30.20) | 27.40 (25.40, 29.70) | 27.60 (25.50, 29.80) | 28.00 (25.90, 30.20)*# | 28.20 (26.10, 30.50)*#△ | 28.40 (26.10, 30.80)*#△ | 28.90 (26.90, 31.35)*#△▲☆ | 28.30 (26.50, 31.20)*#△ | 290.87 | <0.001 |
| ALT, U/L | Male | 23.00 (18.00, 32.00) | 20.00 (15.00, 28.00)a | 23.00 (18.00, 33.00)a* | 24.00 (20.00, 34.00)a*# | 24.00 (19.00, 32.00)a*#△ | 21.00 (17.00, 28.00)a*#△▲ | 19.00 (15.00, 25.00)a*#△▲☆ | 16.00 (12.00, 21.00)*#△▲☆★ | 1819.44 | <0.001 |
|  | Female | 15.00 (11.00, 20.00) | 12.00 (10.00, 16.00) | 13.00 (10.00, 18.00)* | 15.00 (11.00, 19.00)*# | 17.00 (13.00, 23.00)*#△ | 18.00 (14.00, 24.00)*#△▲ | 17.00 (13.00, 22.00)*#△☆ | 14.00 (11.00, 20.00)*#▲☆★ | 2228.31 | <0.001 |
| AST, U/L | Male | 21.00 (18.00, 25.00) | 20.00 (17.00, 24.00)a | 21.00 (18.00, 25.00)a* | 21.00 (19.00, 26.00)a*# | 21.00 (19.00, 25.00)a*# | 21.00 (19.00, 25.00)*# | 22.00 (19.00, 26.00)a*#△▲☆ | 22.00 (19.00, 25.50)*# | 355.74 | <0.001 |
|  | Female | 18.00 (16.00, 21.00) | 17.00 (15.00, 19.00) | 17.00 (15.00, 20.00) | 18.00 (15.00, 21.00)*# | 20.00 (17.00, 23.00)*#△ | 21.00 (19.00, 25.00)*#△▲ | 21.00 (19.00, 25.00)*#△▲ | 21.00 (18.00, 24.00)*#△▲ | 3459.28 | <0.001 |
| ALT/AST | Male | 1.13 (0.91, 1.42) | 1.06 (0.86, 1.41)a | 1.19 (0.95, 1.52)a* | 1.21 (1.00, 1.50)a* | 1.14 (0.94, 1.40)a*#△ | 1.00 (0.83, 1.24)a*#△▲ | 0.87 (0.71, 1.07)a*#△▲☆ | 0.73 (0.60, 0.91)*#△▲☆★ | 3889.72 | <0.001 |
|  | Female | 0.82 (0.67, 1.00) | 0.75 (0.61, 0.92) | 0.79 (0.65, 1.00)* | 0.83 (0.68, 1.00)*# | 0.87 (0.71, 1.06)*#△ | 0.84 (0.71, 1.00)*#▲ | 0.79 (0.65, 0.95)*△▲☆ | 0.71 (0.59, 0.83)*#△▲☆★ | 585.27 | <0.001 |
| AST/ALT | Male | 0.86 (0.68, 1.08) | 0.90 (0.67, 1.14)a | 0.79 (0.62, 1.00)a* | 0.80 (0.65, 1.00)a* | 0.86 (0.70, 1.06)a#△ | 1.00 (0.81, 1.19)a*#△▲ | 1.12 (0.93, 1.36)a*#△▲☆ | 1.25 (1.00, 1.53)a*#△▲☆★ | 4193.76 | <0.001 |
|  | Female | 1.18 (1.00, 1.43) | 1.27 (1.00, 1.54) | 1.20 (1.00, 1.45)* | 1.17 (1.00, 1.42)*# | 1.13 (0.94, 1.36)*#△ | 1.15 (0.98, 1.38)*#▲ | 1.22 (1.00, 1.47)△▲☆ | 1.36 (1.11, 1.62)*#△▲☆★ | 374.58 | <0.001 |
| ALP, U/L | Male | 70.00 (61.00, 83.00) | 72.00 (61.00, 84.00)a | 69.00 (59.00, 81.00)a* | 70.00 (60.00, 82.00)a*# | 71.00 (61.00, 83.00)a#△ | 71.00 (61.00, 83.00)a#△ | 72.00 (62.00, 84.00)a#△ | 73.00 (62.00, 85.00)#△ | 151.02 | <0.001 |
|  | Female | 60.00 (50.00, 72.00) | 55.00 (47.00, 65.00) | 55.00 (47.00, 66.00) | 56.00 (48.00, 66.00) | 66.00 (55.00, 78.00)*#△ | 78.00 (67.00, 91.00)*#△▲ | 75.00 (67.00, 89.00)*#△▲ | 75.00 (67.00, 88.25)*#△▲ | 4977.33 | <0.001 |
| GGT, U/L | Male | 29.00 (25.00, 34.00) | 28.00 (24.00, 33.00)a | 28.00 (24.00, 33.00)a | 29.00 (25.00, 34.00)a*# | 29.00 (25.00, 34.00)a*#△ | 29.00 (25.00, 34.00)a*#△ | 29.00 (25.00, 34.00)a*# | 28.00 (24.50, 33.00)a▲☆ | 207.1 | <0.001 |
|  | Female | 26.00 (22.00, 30.00) | 26.00 (22.00, 30.00) | 25.00 (22.00, 30.00) | 26.00 (22.00, 30.00)# | 27.00 (23.00, 31.00)*#△ | 27.00 (23.00, 32.00)*#△ | 27.00 (23.00, 32.00)*#△ | 27.00 (22.00, 31.00)# | 369.52 | <0.001 |
| AFU | Male | 3.40 (2.70, 5.40) | 3.40 (2.80, 5.50)a | 3.40 (2.70, 5.50)a | 3.40 (2.60, 5.20)a*# | 3.40 (2.70, 5.30)a | 3.40 (2.60, 5.40)a | 3.90 (3.00, 6.00)*#△▲☆ | 4.00 (3.20, 6.60)*#△▲☆ | 177.32 | <0.001 |
|  | Female | 3.00 (1.90, 4.70) | 3.20 (2.10, 5.10) | 2.90 (1.90, 4.50)* | 2.90 (1.90, 4.60)* | 3.10 (2.00, 4.70)#△ | 3.50 (2.20, 5.30)*#△▲ | 3.70 (2.60, 5.70)*#△▲☆ | 4.50 (2.90, 6.73)*#△▲☆ | 355.77 | <0.001 |
| Urea, mmol/L | Male | 4.70 (4.00, 5.50) | 4.60 (3.90, 5.30)a | 4.60 (3.90, 5.40)a | 4.60 (3.90, 5.40)a | 4.60 (4.00, 5.50)a*△ | 4.90 (4.20, 5.80)a*#△▲ | 5.10 (4.50, 6.00)a*#△▲☆ | 5.30 (4.50, 6.20)a*#△▲☆★ | 925.13 | <0.001 |
|  | Female | 4.20 (3.50, 5.00) | 4.10 (3.40, 4.80) | 4.10 (3.40, 4.80) | 4.10 (3.40, 4.90) | 4.40 (3.70, 5.20)*#△ | 4.80 (4.10, 5.60)*#△▲ | 4.90 (4.20, 5.80)*#△▲ | 5.10 (4.38, 5.80)*#△▲☆ | 1283.73 | <0.001 |
| CREA, umol/L | Male | 85.00 (77.00, 93.00) | 84.00 (77.00, 92.00)a | 85.00 (78.00, 92.00)a | 85.00 (77.00, 93.00)a | 84.00 (76.00, 93.00)a#△ | 84.00 (76.00, 93.00)a# | 85.00 (76.00, 95.00)a*▲☆ | 88.00 (78.00, 99.00)a*#△▲☆★ | 115.85 | <0.001 |
|  | Female | 62.00 (56.00, 68.00) | 61.00 (55.00, 66.00) | 61.00 (55.00, 67.00) | 62.00 (56.00, 68.00)*# | 63.00 (58.00, 69.00)*#△ | 64.00 (59.00, 72.00)*#△▲ | 67.00 (59.00, 75.00)*#△▲☆ | 72.00 (64.00, 81.00)*#△▲☆★ | 877.63 | <0.001 |
| UA, umol/L | Male | 393.00 (347.00, 447.00) | 402.00 (358.00, 453.25)a | 403.00 (353.00, 457.00)a | 397.00 (350.00, 450.00)a*# | 391.00 (345.00, 443.00)a*#△ | 374.00 (327.00, 427.00)a*#△▲ | 374.00 (323.00, 430.00)a*#△▲ | 375.00 (326.00, 436.50)a*#△▲ | 795.27 | <0.001 |
|  | Female | 279.00 (243.00, 319.00) | 286.00 (252.00, 326.00) | 279.00 (243.00, 318.00)* | 269.00 (234.00, 308.00)*# | 283.00 (247.00, 324.00)#△ | 296.00 (258.00, 338.00)*#△▲ | 310.00 (270.00, 360.00)*#△▲☆ | 317.00 (270.75, 384.00)*#△▲☆ | 847.96 | <0.001 |
| TC, mmol/L | Male | 4.70 (4.19, 5.27) | 4.18 (3.72, 4.71)a | 4.57 (4.06, 5.11)a* | 4.76 (4.26, 5.32)a*# | 4.81 (4.31, 5.39)*#△ | 4.82 (4.30, 5.38)a*#△ | 4.68 (4.15, 5.29)a*#△▲☆ | 4.63 (4.06, 5.20)a*△▲☆ | 1718.32 | <0.001 |
|  | Female | 4.45 (3.95, 5.02) | 4.01 (3.59, 4.46) | 4.17 (3.74, 4.65)* | 4.39 (3.95, 4.89)*# | 4.82 (4.32, 5.36)*#△ | 5.12 (4.57, 5.70)*#△▲ | 5.05 (4.45, 5.62)*#△▲ | 4.84 (4.24, 5.52)*#△☆ | 4795.51 | <0.001 |
| TG, mmol/L | Male | 1.55 (1.20, 2.23) | 1.19 (0.89, 1.69)a | 1.48 (1.13, 2.15)a* | 1.64 (1.31, 2.35)a*# | 1.69 (1.34, 2.37)a*#△ | 1.50 (1.15, 2.10)*△▲ | 1.40 (1.04, 1.87)a*#△▲☆ | 1.21 (0.94, 1.63)a#△▲☆★ | 1967.22 | <0.001 |
|  | Female | 1.11 (0.81, 1.55) | 0.83 (0.65, 1.10) | 0.93 (0.71, 1.28)* | 1.09 (0.81, 1.49)*# | 1.33 (0.98, 1.80)*#△ | 1.50 (1.12, 2.04)*#△▲ | 1.51 (1.16, 2.00)*#△▲ | 1.45 (1.09, 1.88)*#△ | 3921.68 | <0.001 |
| HDL, mmol/L | Male | 1.22 (1.06, 1.41) | 1.26 (1.10, 1.45)a | 1.21 (1.05, 1.40)a* | 1.21 (1.05, 1.40)a* | 1.20 (1.05, 1.39)a* | 1.27 (1.10, 1.47)a#△▲ | 1.31 (1.11, 1.51)a*#△▲☆ | 1.34 (1.15, 1.57)a*#△▲☆★ | 618.97 | <0.001 |
|  | Female | 1.46 (1.28, 1.68) | 1.49 (1.31, 1.72) | 1.47 (1.28, 1.69)* | 1.45 (1.27, 1.68)*# | 1.44 (1.27, 1.66)*# | 1.43 (1.27, 1.67)*# | 1.47 (1.29, 1.68) | 1.40 (1.25, 1.64)* | 64.49 | <0.001 |
| LDL, mmol/L | Male | 2.68 (2.23, 3.18) | 2.42 (1.98, 2.84)a | 2.62 (2.18, 3.10)a* | 2.71 (2.27, 3.21)a*# | 2.73 (2.28, 3.24)a*# | 2.78 (2.30, 3.27)a*#△ | 2.71 (2.22, 3.21)a*#☆ | 2.63 (2.18, 3.12)*△▲☆ | 628.71 | <0.001 |
|  | Female | 2.50 (2.07, 2.98) | 2.15 (1.80, 2.53) | 2.28 (1.91, 2.70)* | 2.46 (2.07, 2.89)*# | 2.76 (2.34, 3.25)*#△ | 2.98 (2.51, 3.50)*#△▲ | 2.92 (2.43, 3.47)*#△▲ | 2.72 (2.26, 3.24)*#△☆★ | 3622.54 | <0.001 |
| VLDL, mmol/L | Male | 0.68 (0.53, 0.98) | 0.52 (0.39, 0.74)a | 0.65 (0.50, 0.94)a* | 0.72 (0.58, 1.03)a*# | 0.74 (0.59, 1.04)a*#△ | 0.66 (0.51, 0.92)*△▲ | 0.62 (0.46, 0.82)a*#△▲☆ | 0.53 (0.41, 0.72)a#△▲☆★ | 1975.58 | <0.001 |
|  | Female | 0.49 (0.36, 0.68) | 0.37 (0.29, 0.48) | 0.41 (0.31, 0.56)* | 0.48 (0.36, 0.66)*# | 0.59 (0.43, 0.79)*#△ | 0.66 (0.49, 0.90)*#△▲ | 0.66 (0.51, 0.88)*#△▲ | 0.64 (0.48, 0.83)*#△ | 3919.33 | <0.001 |
| APOA1, g/L | Male | 1.48 (1.32, 1.66) | 1.43 (1.29, 1.59)a | 1.45 (1.30, 1.62)a* | 1.48 (1.32, 1.66)a*# | 1.49 (1.33, 1.67)a*#△ | 1.53 (1.38, 1.72)a*#△▲ | 1.54 (1.38, 1.73)a*#△▲ | 1.55 (1.38, 1.75)a*#△▲ | 704.88 | <0.001 |
|  | Female | 1.61 (1.45, 1.80) | 1.56 (1.41, 1.74) | 1.58 (1.42, 1.76) | 1.61 (1.45, 1.79)*# | 1.64 (1.48, 1.83)*#△ | 1.68 (1.52, 1.88)*#△▲ | 1.70 (1.53, 1.88)*#△▲ | 1.67 (1.49, 1.89)*#△ | 559.19 | <0.001 |
| APOB100, g/L | Male | 0.96 (0.82, 1.12) | 0.81 (0.68, 0.96)a | 0.92 (0.78, 1.08)a* | 0.98 (0.85, 1.13)a*# | 1.00 (0.87, 1.15)a*#△ | 0.99 (0.85, 1.14)*#▲ | 0.95 (0.80, 1.10)a*#△▲☆ | 0.91 (0.76, 1.06)*△▲☆★ | 2092.89 | <0.001 |
|  | Female | 0.82 (0.69, 0.98) | 0.68 (0.59, 0.80) | 0.74 (0.63, 0.87)* | 0.81 (0.69, 0.95)*# | 0.92 (0.79, 1.07)*#△ | 1.01 (0.86, 1.16)*#△▲ | 0.99 (0.84, 1.14)*#△▲ | 0.94 (0.78, 1.09)*#△☆ | 5341.33 | <0.001 |
| LP (a), mg/L | Male | 92.00 (35.00, 180.00) | 73.00 (31.00, 158.25)a | 80.00 (32.00, 166.00)a | 92.00 (35.00, 178.00)a*# | 92.00 (35.00, 176.00)a*# | 92.00 (43.00, 207.00)a*#△▲ | 105.00 (51.00, 222.00)*#△▲☆ | 111.00 (58.00, 225.00)*#△▲☆ | 422.67 | <0.001 |
|  | Female | 92.00 (47.00, 206.00) | 92.00 (39.00, 191.00) | 92.00 (42.00, 192.00) | 92.00 (46.00, 203.75)*# | 95.00 (53.00, 216.00)*#△ | 108.00 (66.00, 242.00)*#△▲ | 121.00 (64.50, 261.50)*#△▲ | 105.00 (61.50, 252.50)*# | 197.95 | <0.001 |
| GLU, mmol/L | Male | 4.80 (4.60, 5.20) | 4.60 (4.30, 4.90) | 4.70 (4.40, 5.00)a* | 4.80 (4.60, 5.20)a*# | 4.90 (4.70, 5.30)a*#△ | 5.00 (4.80, 5.40)*#△▲ | 5.00 (4.80, 5.40)*#△▲ | 5.00 (4.80, 5.40)*#△▲ | 3178.01 | <0.001 |
|  | Female | 4.80 (4.50, 5.10) | 4.60 (4.40, 4.90) | 4.70 (4.40, 5.00)* | 4.80 (4.50, 5.10)*# | 4.90 (4.60, 5.20)*#△ | 5.00 (4.80, 5.40)*#△▲ | 5.10 (4.80, 5.50)*#△▲ | 5.00 (4.80, 5.40)*#△▲ | 2536.09 | <0.001 |
| WBC, 10^9/L | Male | 6.33 (5.44, 7.38) | 6.22 (5.31, 7.19) | 6.33 (5.46, 7.36)* | 6.45 (5.55, 7.52)*# | 6.27 (5.35, 7.25)#△ | 6.31 (5.41, 7.31)△ | 6.36 (5.52, 7.36)*▲ | 6.30 (5.42, 7.39)△ | 199.45 | <0.001 |
|  | Female | 6.33 (5.43, 7.33) | 6.14 (5.26, 7.11) | 6.33 (5.46, 7.38)* | 6.40 (5.52, 7.42)* | 6.21 (5.31, 7.19)#△ | 6.24 (5.40, 7.23)△ | 6.36 (5.46, 7.49)*▲ | 6.28 (5.40, 7.20) | 150.01 | <0.001 |
| NEUT, % | Male | 56.60 (51.30, 61.70) | 56.60 (51.30, 61.56) | 56.70 (51.44, 61.90) | 56.60 (51.37, 61.70) | 56.60 (51.30, 61.80) | 56.24 (50.90, 61.40)# | 56.30 (51.00, 61.50) | 56.00 (51.05, 61.00)# | 24.68 | 0.001 |
|  | Female | 56.70 (51.54, 62.00) | 56.70 (52.00, 61.90) | 56.84 (51.74, 62.30)* | 56.70 (51.60, 61.90)*# | 56.60 (51.40, 62.09)*#△ | 56.60 (51.20, 61.70)*#△ | 56.50 (50.90, 62.10)*#△▲☆ | 56.05 (50.55, 61.23)*#△▲☆★ | 12.98 | 0.03 |
| LYMPH, % | Male | 35.14 (30.40, 40.10) | 35.00 (30.20, 39.96) | 35.14 (30.37, 40.10) | 35.40 (30.70, 40.40)# | 34.80 (30.10, 39.80)#△ | 35.40 (30.70, 40.30)▲ | 34.90 (30.40, 39.90) | 35.14 (30.90, 40.00) | 61.22 | <0.001 |
|  | Female | 35.14 (30.40, 40.20) | 34.80 (30.24, 39.70) | 35.14 (30.10, 40.10) | 35.40 (30.80, 40.50)*# | 35.00 (30.10, 39.90)△ | 35.34 (30.84, 40.30) | 34.60 (30.02, 39.90)△ | 35.07 (30.58, 40.13) | 42.27 | <0.001 |
| MONO, % | Male | 5.20 (4.40, 6.20) | 5.54 (4.80, 6.60)a | 5.20 (4.30, 6.20)a* | 5.10 (4.20, 6.00)a*# | 5.30 (4.50, 6.30)a*#△ | 5.20 (4.40, 6.10)a*△▲ | 5.54 (4.80, 6.50)#△▲☆ | 5.60 (4.90, 6.52)#△▲☆ | 908.5 | <0.001 |
|  | Female | 5.10 (4.30, 6.00) | 5.40 (4.70, 6.30) | 5.10 (4.20, 6.00)* | 4.90 (4.04, 5.70)*# | 5.20 (4.50, 6.10)*#△ | 5.10 (4.30, 5.90)*△▲ | 5.50 (4.80, 6.40)#△▲☆ | 5.70 (5.00, 6.73)*#△▲☆ | 717.46 | <0.001 |
| EO, % | Male | 1.70 (1.10, 2.60) | 1.70 (1.10, 2.50) | 1.70 (1.10, 2.60) | 1.70 (1.10, 2.60)* | 1.70 (1.20, 2.70)*#△ | 1.70 (1.20, 2.70)*# | 1.70 (1.20, 2.80)*# | 1.70 (1.10, 2.70) | 61.1 | <0.001 |
|  | Female | 1.70 (1.10, 2.60) | 1.70 (1.10, 2.50) | 1.70 (1.10, 2.60) | 1.70 (1.10, 2.60) | 1.70 (1.20, 2.70)# | 1.70 (1.10, 2.70) | 1.70 (1.20, 2.80) | 1.70 (1.20, 2.80) | 25.42 | 0.001 |
| RBC, 10^12/L | Male | 5.13 (4.79, 5.46) | 5.05 (4.70, 5.38)a | 5.15 (4.80, 5.48)a* | 5.10 (4.76, 5.43)a*# | 5.16 (4.83, 5.48)a*△ | 5.09 (4.77, 5.41)*#▲ | 5.19 (4.87, 5.54)*#△▲☆ | 5.19 (4.86, 5.54)*△☆ | 283.22 | <0.001 |
|  | Female | 5.09 (4.76, 5.42) | 4.98 (4.66, 5.31) | 5.10 (4.77, 5.43)* | 5.07 (4.73, 5.39)*# | 5.13 (4.79, 5.46)*#△ | 5.08 (4.77, 5.39)*▲ | 5.25 (4.90, 5.54)*#△▲☆ | 5.23 (4.92, 5.56)*#△▲☆ | 267.31 | <0.001 |
| HB, g/L | Male | 158.00 (145.00, 168.00) | 157.00 (144.00, 168.00)a | 159.00 (146.00, 168.00)a* | 157.00 (144.00, 167.00)a# | 158.00 (146.00, 169.00)a*△ | 155.00 (143.00, 165.00)*#△▲ | 160.00 (146.00, 170.00)*△▲☆ | 160.00 (147.00, 170.00)*△☆ | 257.69 | <0.001 |
|  | Female | 156.00 (144.00, 167.00) | 153.00 (143.00, 166.00) | 157.00 (145.00, 167.00)* | 155.00 (143.00, 166.00)# | 157.00 (144.00, 168.00)*△ | 154.00 (142.00, 165.00)#△▲ | 160.00 (147.00, 171.00)*#△▲☆ | 161.00 (147.75, 170.00)*#△▲☆ | 159.01 | <0.001 |
| HCT, % | Male | 0.47 (0.44, 0.50) | 0.46 (0.43, 0.49)a | 0.47 (0.44, 0.50)a* | 0.47 (0.43, 0.49)a*# | 0.47 (0.44, 0.50)a*△ | 0.46 (0.43, 0.49)#▲ | 0.47 (0.44, 0.50)*△☆ | 0.48 (0.44, 0.50)*△☆ | 267.51 | <0.001 |
|  | Female | 0.46 (0.43, 0.49) | 0.45 (0.42, 0.48) | 0.47 (0.44, 0.49)* | 0.46 (0.43, 0.49)*# | 0.47 (0.44, 0.50)*△ | 0.46 (0.43, 0.49)*#▲ | 0.47 (0.44, 0.50)*#△☆ | 0.48 (0.44, 0.50)*#△▲☆ | 240.79 | <0.001 |
| MCV, fL | Male | 91.00 (88.70, 93.50) | 91.10 (89.00, 93.60) | 91.10 (88.80, 93.50) | 91.10 (88.70, 93.60) | 91.00 (88.60, 93.60)a | 91.00 (88.60, 93.50) | 90.50 (88.10, 92.70)*#△▲☆ | 91.00 (88.80, 93.60)★ | 78.96 | <0.001 |
|  | Female | 91.00 (88.60, 93.40) | 91.00 (88.60, 93.40) | 91.10 (88.70, 93.50) | 91.00 (88.70, 93.40) | 90.90 (88.30, 93.30)#△ | 90.80 (88.50, 93.10)# | 89.80 (87.60, 92.20)*#△▲☆ | 90.70 (88.40, 93.00)★ | 96.9 | <0.001 |
| MCH, pg | Male | 30.60 (29.80, 31.50) | 30.90 (30.10, 31.80) | 30.60 (29.80, 31.50)* | 30.60 (29.80, 31.50)* | 30.60 (29.70, 31.40)a*#△ | 30.40 (29.60, 31.30)*#△▲ | 30.60 (29.70, 31.40)*#☆ | 30.60 (29.80, 31.50)*☆ | 331.47 | <0.001 |
|  | Female | 30.60 (29.80, 31.40) | 30.90 (30.10, 31.70) | 30.60 (29.80, 31.50)* | 30.60 (29.80, 31.50)* | 30.50 (29.60, 31.30)*#△ | 30.40 (29.50, 31.10)*#△▲ | 30.40 (29.60, 31.30)*#△ | 30.50 (29.70, 31.43)* | 326.19 | <0.001 |
| MCHC, g/L | Male | 336.00 (330.00, 341.00) | 339.00 (333.00, 344.00) | 336.00 (330.00, 342.00)* | 336.00 (330.00, 341.00)*# | 335.00 (329.00, 341.00)*#△ | 333.00 (327.00, 339.00)*#△▲ | 337.00 (332.00, 343.00)*#△▲☆ | 336.00 (330.00, 341.00)*☆★ | 904.74 | <0.001 |
|  | Female | 336.00 (330.00, 342.00) | 339.00 (334.00, 345.00) | 336.00 (330.00, 342.00)* | 336.00 (330.00, 341.00)*# | 335.00 (329.00, 341.00)*#△ | 333.00 (327.00, 340.00)*#△▲ | 338.00 (332.00, 344.00)*#△▲☆ | 336.00 (330.00, 342.00)*☆ | 603.87 | <0.001 |
| RDW, % | Male | 13.00 (12.60, 13.50) | 13.10 (12.70, 13.50)a | 13.00 (12.60, 13.40)a* | 13.00 (12.70, 13.50)a# | 13.00 (12.60, 13.50)a# | 13.10 (12.70, 13.60)*#△▲ | 12.90 (12.50, 13.20)*#△▲☆ | 12.80 (12.40, 13.20)*#△▲☆ | 489.52 | <0.001 |
|  | Female | 13.10 (12.70, 13.50) | 13.10 (12.80, 13.50) | 13.00 (12.70, 13.50)* | 13.10 (12.70, 13.50)* | 13.10 (12.70, 13.58)*#△ | 13.20 (12.80, 13.60)#△▲ | 12.90 (12.50, 13.30)*#△▲☆ | 12.70 (12.40, 13.10)*#△▲☆★ | 253.95 | <0.001 |
| PLT, 10^9/L | Male | 216.00 (184.00, 252.00) | 215.00 (183.00, 251.00)a | 216.00 (184.00, 251.00)a | 219.00 (187.00, 255.00)*# | 211.00 (178.00, 245.00)*#△ | 220.00 (187.00, 257.00)*#▲ | 221.00 (189.00, 257.00)*#▲ | 220.00 (187.00, 257.00)▲ | 315.09 | <0.001 |
|  | Female | 215.00 (183.00, 250.00) | 209.00 (179.00, 242.75) | 213.00 (181.00, 247.00) | 219.00 (186.00, 255.00)*# | 209.00 (178.00, 245.00)#△ | 223.00 (189.00, 259.00)*#▲ | 223.00 (191.00, 257.50)*#▲ | 215.50 (186.75, 249.00) | 251.98 | <0.001 |
| MPV, fL | Male | 11.40 (10.60, 12.20) | 11.80 (11.10, 12.60)a | 11.50 (10.80, 12.30)a | 11.50 (10.80, 12.30)a | 11.20 (10.50, 12.00) | 11.00 (10.30, 11.80) | 11.00 (10.30, 11.80) | 11.10 (10.40, 11.90) | 1607.9 | <0.001 |
|  | Female | 11.50 (10.80, 12.30) | 12.00 (11.30, 12.80) | 11.60 (10.90, 12.40) | 11.60 (10.90, 12.40) | 11.30 (10.50, 12.10) | 11.00 (10.30, 11.90) | 11.10 (10.40, 11.70) | 11.10 (10.40, 11.80) | 1260.25 | <0.001 |
| PCT, % | Male | 2.50 (2.14, 2.80) | 2.50 (2.24, 2.90)a | 2.50 (2.20, 2.80) | 2.50 (2.20, 2.90)*# | 2.40 (2.10, 2.70)*#△ | 2.40 (2.10, 2.70)*#▲ | 2.50 (2.10, 2.80)*#▲ | 2.44 (2.10, 2.80)▲ | 868.55 | <0.001 |
|  | Female | 2.50 (2.20, 2.80) | 2.50 (2.20, 2.80) | 2.50 (2.20, 2.80) | 2.50 (2.20, 2.90)*# | 2.40 (2.10, 2.70)#△ | 2.40 (2.14, 2.80)*#▲ | 2.44 (2.17, 2.80)*#▲ | 2.40 (2.10, 2.70) | 573.9 | <0.001 |
| PDW, fL | Male | 14.10 (12.60, 15.80) | 14.40 (12.90, 16.30)a | 14.10 (12.70, 15.90)a* | 14.10 (12.70, 15.90)a* | 14.10 (12.60, 15.80)* | 13.50 (12.20, 15.35)*#△▲ | 13.60 (12.30, 15.30)*#△▲ | 13.80 (12.30, 15.40)*#△▲ | 372.16 | <0.001 |
|  | Female | 14.10 (12.80, 16.00) | 14.70 (13.30, 16.70) | 14.30 (12.90, 16.20)* | 14.10 (12.80, 16.10)* | 14.10 (12.70, 15.90)*# | 13.60 (12.20, 15.30)*#△▲ | 13.70 (12.45, 15.20)*#△▲ | 14.00 (12.70, 15.60)* | 311.04 | <0.001 |
| P-LCR, % | Male | 36.20 (30.00, 42.80) | 39.80 (33.20, 46.63)a | 37.10 (31.00, 43.51)a* | 37.30 (31.20, 43.71)a* | 35.20 (29.20, 41.80)*#△ | 33.10 (27.40, 40.00)*#△▲ | 33.40 (27.60, 39.60)*#△▲ | 33.80 (27.85, 40.25)*#△▲ | 1447.49 | <0.001 |
|  | Female | 37.10 (31.00, 43.81) | 41.60 (35.40, 47.71) | 38.10 (32.00, 44.61)* | 37.90 (31.90, 44.21)* | 35.50 (29.40, 42.10)*#△ | 33.50 (27.40, 40.30)*#△▲ | 33.70 (28.15, 39.10)*#△▲ | 34.30 (28.30, 39.83)*#△ | 1160.18 | <0.001 |

For the same parameter within the same age group, after Bonferroni correction a P < 0.05 vs. females.

For the same parameter, after Bonferroni correction, compared with the 18-25 age group, * P < 0.05; compared with the 26-35 age group, # P < 0.05; compared with the 36-45 age group, △ P < 0.05; compared with the 46-55 age group, ▲ P < 0.05; compared with the 56-65 age group, ☆ P < 0.05; and compared with the 66-75 age group, ★ P < 0.05.

TBIL: total bilirubin; DBIL: direct bilirubin; IBIL: indirect bilirubin; TP: total protein; ALB: albumin; GLB: globulin; ALT: alanine aminotransferase; AST: aspartate aminotransferase; ALT/AST: alanine aminotransferase/aspartate aminotransferase ratio; AST/ALT: aspartate aminotransferase/alanine aminotransferase ratio; ALP: alkaline phosphatase; GGT: γ-glutamyl transpeptidase; AFU: α-L-fucosidase; Urea: Urea; CREA: creatinine; UA: uric acid; TC: total cholesterol; TG: triglyceride; HDL: high-density lipoprotein cholesterol; LDL: low-density lipoprotein cholesterol; VLDL: Very low-density lipoprotein cholesterol; ApoA1: apolipoprotein A1; ApoB100: apolipoprotein B100; Lp(a): lipoprotein(a); GLU: glucose; WBC: white blood cell count; NEUT: neutrophil percentage; LYM: lymphocyte percentage; MONO: monocyte percentage; EO: eosinophil percentage; RBC: red blood cell count; Hb: hemoglobin; HCT: hematocrit; MCV: mean corpuscular volume; MCH: mean corpuscular hemoglobin; MCHC: mean corpuscular hemoglobin concentration; PLT: patelet count; RDW: red cell distribution width; PDW: platelet distribution width; MPV: mean platelet volume; PCT: plateletcrit; P-LCR: platelet–large cell ratio.
