# Supplementary material for: A cross-sectional study of routine hematological and biochemical parameters with age- and sex-specific analyses in mid-to-high-altitude regions of southwestern China
Source: Front Public Health. 2026 Oct 28;14:1949654. doi: 10.3389/fpubh.2026.1949654 (PMC13752126; doi:10.3389/fpubh.2026.1949654)
Supplement: Supplementary file 2 [file Table_2.docx]

Table S2: Relative median differences (RMD) between males and females across age groups.

| Variables | Different age groups | | | | | | |
| --- | --- | --- | --- | --- | --- | --- | --- |
|  | 18-25 (years) | 26-35 (years) | 36-45 (years) | 46-55 (years) | 56-65 (years) | 66-75 (years) | >75 (years) |
| TBIL, umol/L | 21.05 | 17.70 | 16.81 | 13.66 | 12.71 | 14.17 | 15.00 |
| DBIL, umol/L | 26.67 | 17.24 | 18.52 | 28.00 | 32.00 | 25.93 | 24.14 |
| IBIL, umol/L | 19.28 | 16.67 | 15.12 | 10.34 | 7.53 | 8.60 | 8.60 |
| TP, g/L | 0.13 | -0.13 | -0.13 | -1.07 | -1.20 | -0.66 | 0.81 |
| ALB, g/L | 3.51 | 3.33 | 3.62 | 2.35 | 0.85 | 0.65 | 0.77 |
| GLB, g/L | -5.84 | -6.16 | -6.43 | -6.74 | -4.93 | -2.77 | 0.71 |
| ALT, U/L | 66.67 | 76.92 | 60.00 | 41.18 | 16.67 | 11.76 | 14.29 |
| AST, U/L | 17.65 | 23.53 | 16.67 | 5.00 | 0.00 | 4.76 | 4.76 |
| ALT/AST | 41.33 | 50.63 | 45.78 | 31.03 | 19.05 | 10.13 | 3.55 |
| AST/ALT | -29.13 | -34.17 | -31.62 | -23.89 | -13.04 | -8.20 | -8.09 |
| ALP, U/L | 30.91 | 25.45 | 25.00 | 7.58 | -8.97 | -4.00 | -2.67 |
| GGT, U/L | 7.69 | 12.00 | 11.54 | 7.41 | 7.41 | 7.41 | 3.70 |
| AFU | 6.25 | 17.24 | 17.24 | 9.68 | -2.86 | 5.41 | -11.11 |
| Urea, mmol/L | 12.2 | 12.20 | 12.20 | 4.55 | 2.08 | 4.08 | 3.92 |
| CREA, umol/L | 37.7 | 39.34 | 37.10 | 33.33 | 31.25 | 26.87 | 22.22 |
| UA, umol/L | 40.56 | 44.44 | 47.58 | 38.16 | 26.35 | 20.65 | 18.30 |
| TC, mmol/L | 4.24 | 9.59 | 8.43 | -0.21 | -5.86 | -7.33 | -4.24 |
| TG, mmol/L | 43.37 | 59.14 | 50.46 | 27.07 | 0.00 | -7.28 | -16.26 |
| HDL, mmol/L | -15.44 | -17.69 | -16.55 | -16.67 | -11.19 | -10.88 | -4.29 |
| LDL, mmol/L | 12.33 | 14.91 | 10.16 | -1.09 | -6.71 | -7.19 | -3.31 |
| VLDL, mmol/L | 40.54 | 58.54 | 50.00 | 25.42 | 0.00 | -6.06 | -16.54 |
| APOA1, g/L | -8.33 | -8.23 | -8.07 | -9.15 | -8.93 | -9.41 | -6.91 |
| APOB100, g/L | 19.12 | 24.32 | 20.99 | 8.70 | -1.98 | -4.04 | -3.19 |
| LP (a), mg/L | -20.65 | -13.04 | 0.00 | -3.16 | -14.81 | -13.22 | 5.71 |
| GLU, mmol/L | 0 | 0.00 | 0.00 | 0.00 | 0.00 | -1.96 | 0.00 |
| WBC, 10^9/L | 1.3 | 0.00 | 0.78 | 0.97 | 1.12 | 0.00 | 0.40 |
| NEUT, % | -0.18 | -0.25 | -0.18 | 0.00 | -0.64 | -0.35 | -0.09 |
| LYMPH, % | 0.57 | 0.00 | 0.00 | -0.57 | 0.17 | 0.87 | 0.20 |
| MONO, % | 2.59 | 1.96 | 4.08 | 1.92 | 1.96 | 0.73 | -1.75 |
| EO, % | 0 | 0.00 | 0.00 | 0.00 | 0.00 | 0.00 | 0.00 |
| RBC, 10^12/L | 1.41 | 0.98 | 0.59 | 0.58 | 0.20 | -1.14 | -0.67 |
| HB, g/L | 2.61 | 1.27 | 1.29 | 0.64 | 0.65 | 0.00 | -0.62 |
| HCT, % | 1.55 | 1.07 | 1.08 | 1.07 | 0.87 | 0.00 | -1.04 |
| MCV, fL | 0.11 | 0.00 | 0.11 | 0.11 | 0.22 | 0.78 | 0.33 |
| MCH, pg | 0 | 0.00 | 0.00 | 0.33 | 0.00 | 0.66 | 0.33 |
| MCHC, g/L | 0 | 0.00 | 0.00 | 0.00 | 0.00 | -0.30 | 0.00 |
| RDW, % | 0 | 0.00 | -0.76 | -0.76 | -0.76 | 0.00 | 0.79 |
| PLT, 10^9/L | 2.87 | 1.41 | 0.00 | 0.96 | -1.35 | -0.90 | 2.09 |
| MPV, fL | -1.67 | -0.86 | -0.86 | -0.88 | 0.00 | -0.90 | 0.00 |
| PCT, % | 0 | 0.00 | 0.00 | 0.00 | 0.00 | 2.46 | 1.67 |
| PDW, fL | -2.04 | -1.40 | 0.00 | 0.00 | -0.74 | -0.73 | -1.43 |
| P-LCR, % | -4.33 | -2.62 | -1.58 | -0.85 | -1.19 | -0.89 | -1.46 |

The value is positive when it indicates that men are more than women; the value is negative when it shows that men are less than women. When |RMD| is greater than 10%, it has clinical significance. RMD: relative median difference.
