# Supplementary material for: A cross-sectional study of routine hematological and biochemical parameters with age- and sex-specific analyses in mid-to-high-altitude regions of southwestern China
Source: Front Public Health. 2026 Oct 28;14:1949654. doi: 10.3389/fpubh.2026.1949654 (PMC13752126; doi:10.3389/fpubh.2026.1949654)
Supplement: Supplementary file 3 [file Table_3.docx]

Table S3: Correlation of indicators for women at different age groups (Bonferroni correction).

| Sex-Age | Variables | R | Original P | Correction P | Correction significance |
| --- | --- | --- | --- | --- | --- |
| Female- 18-25 (years) | TG-UA | 0.25 | 0.00 | 0.00 | *** |
| Female- 26-35 (years) | TG-UA | 0.26 | 0.00 | 0.00 | *** |
| Female- 36-45 (years) | TG-UA | 0.31 | 0.00 | 0.00 | *** |
| Female- 46-55 (years) | TG-UA | 0.29 | 0.00 | 0.00 | *** |
| Female- 56-65 (years) | TG-UA | 0.29 | 0.00 | 0.00 | *** |
| Female- 66-75 (years) | TG-UA | 0.25 | 0.00 | 0.00 | *** |
| Female- >75 (years) | TG-UA | 0.26 | 0.00 | 0.00 | ** |
| Female- 18-25 (years) | VLDL-UA | 0.25 | 0.00 | 0.00 | *** |
| Female- 26-35 (years) | VLDL-UA | 0.26 | 0.00 | 0.00 | *** |
| Female- 36-45  (years) | VLDL-UA | 0.31 | 0.00 | 0.00 | *** |
| Female- 46-55 (years) | VLDL-UA | 0.29 | 0.00 | 0.00 | *** |
| Female- 56-65 (years) | VLDL-UA | 0.29 | 0.00 | 0.00 | *** |
| Female- 66-75 (years) | VLDL-UA | 0.25 | 0.00 | 0.00 | *** |
| Female- >75 (years) | VLDL-UA | 0.26 | 0.00 | 0.00 | ** |
| Female- 18-25 (years) | APOB100-DBIL | -0.19 | 0.00 | 0.00 | *** |
| Female- 26-35 (years) | APOB100-DBIL | -0.25 | 0.00 | 0.00 | *** |
| Female- 36-45 (years) | APOB100-DBIL | -0.25 | 0.00 | 0.00 | *** |
| Female- 46-55 (years) | APOB100-DBIL | -0.24 | 0.00 | 0.00 | *** |
| Female- 56-65 (years) | APOB100-DBIL | -0.24 | 0.00 | 0.00 | *** |
| Female- 66-75 (years) | APOB100-DBIL | -0.24 | 0.00 | 0.00 | *** |
| Female- >75 (years) | APOB100-DBIL | -0.31 | 0.00 | 0.00 | *** |

R: Spearman correlation coefficient; Original P: original P value; Correction P: Bonferroni-corrected P value. * adjusted P<0.05; ** adjusted P<0.01; *** adjusted P<0.001.

TG: Triglyceride; UA: Uric acid; VLDL: Very low-density lipoprotein cholesterol; ApoB100: Apolipoprotein B100; DBIL: Direct bilirubin.
