# Supplementary material for: A cross-sectional study of routine hematological and biochemical parameters with age- and sex-specific analyses in mid-to-high-altitude regions of southwestern China
Source: Front Public Health. 2026 Oct 28;14:1949654. doi: 10.3389/fpubh.2026.1949654 (PMC13752126; doi:10.3389/fpubh.2026.1949654)
Supplement: Supplementary file 4 [file Table_4.docx]

Table S4: Correlation of indicators for men at different age groups (Bonferroni correction).

| Sex-Age | Variables | R | Original P | Correction P | Correction significance | Sex-Age | Variables | R | Original P | Correction P | Correction significance |
| --- | --- | --- | --- | --- | --- | --- | --- | --- | --- | --- | --- |
| Male- 18-25 (years) | VLDL-UA | 0.28 | 0.00 | 0.00 | *** | Male- 18-25 (years) | TG-ALT/AST | 0.34 | 0.00 | 0.00 | *** |
| Male- 26-35 (years) | VLDL-UA | 0.28 | 0.00 | 0.00 | *** | Male- 26-35 (years) | TG-ALT/AST | 0.31 | 0.00 | 0.00 | *** |
| Male- 36-45 (years) | VLDL-UA | 0.22 | 0.00 | 0.00 | *** | Male- 36-45 (years) | TG-ALT/AST | 0.24 | 0.00 | 0.00 | *** |
| Male- 46-55 (years) | VLDL-UA | 0.20 | 0.00 | 0.00 | *** | Male- 46-55 (years) | TG-ALT/AST | 0.21 | 0.00 | 0.00 | *** |
| Male- 56-65 (years) | VLDL-UA | 0.25 | 0.00 | 0.00 | *** | Male- 56-65 (years) | TG-ALT/AST | 0.24 | 0.00 | 0.00 | *** |
| Male- 66-75 (years) | VLDL-UA | 0.22 | 0.00 | 0.00 | *** | Male- 66-75 (years) | TG-ALT/AST | 0.20 | 0.00 | 0.00 | *** |
| Male- >75 (years) | VLDL-UA | 0.30 | 0.00 | 0.00 | *** | Male- >75 (years) | TG-ALT/AST | 0.24 | 0.00 | 0.00 | *** |
| Male- 18-25 (years) | VLDL-AST/ALT | -0.39 | 0.00 | 0.00 | *** | Male- 18-25 (years) | TG-ALT | 0.33 | 0.00 | 0.00 | *** |
| Male- 26-35 (years) | VLDL-AST/ALT | -0.35 | 0.00 | 0.00 | *** | Male- 26-35 (years) | TG-ALT | 0.29 | 0.00 | 0.00 | *** |
| Male- 36-45 (years) | VLDL-AST/ALT | -0.27 | 0.00 | 0.00 | *** | Male- 36-45 (years) | TG-ALT | 0.21 | 0.00 | 0.00 | *** |
| Male- 46-55 (years) | VLDL-AST/ALT | -0.22 | 0.00 | 0.00 | *** | Male- 46-55 (years) | TG-ALT | 0.18 | 0.00 | 0.00 | *** |
| Male- 56-65 (years) | VLDL-AST/ALT | -0.24 | 0.00 | 0.00 | *** | Male- 56-65 (years) | TG-ALT | 0.18 | 0.00 | 0.00 | *** |
| Male- 66-75 (years) | VLDL-AST/ALT | -0.20 | 0.00 | 0.00 | *** | Male- 66-75 (years) | TG-ALT | 0.14 | 0.00 | 0.00 | *** |
| Male- >75 (years) | VLDL-AST/ALT | -0.16 | 0.00 | 0.00 | *** | Male- >75 (years) | TG-ALT | 0.15 | 0.00 | 0.00 | *** |
| Male- 18-25 (years) | VLDL-ALT/AST | 0.34 | 0.00 | 0.00 | *** | Male- 18-25 (years) | HDL-AST/ALT | 0.32 | 0.00 | 0.00 | *** |
| Male- 26-35 (years) | VLDL-ALT/AST | 0.31 | 0.00 | 0.00 | *** | Male- 26-35 (years) | HDL-AST/ALT | 0.34 | 0.00 | 0.00 | *** |
| Male- 36-45 (years) | VLDL-ALT/AST | 0.24 | 0.00 | 0.00 | *** | Male- 36-45 (years) | HDL-AST/ALT | 0.31 | 0.00 | 0.00 | *** |
| Male- 46-55 (years) | VLDL-ALT/AST | 0.21 | 0.00 | 0.00 | *** | Male- 46-55 (years) | HDL-AST/ALT | 0.26 | 0.00 | 0.00 | *** |
| Male- 56-65 (years) | VLDL-ALT/AST | 0.24 | 0.00 | 0.00 | *** | Male- 56-65 (years) | HDL-AST/ALT | 0.24 | 0.00 | 0.00 | *** |
| Male- 66-75 (years) | VLDL-ALT/AST | 0.20 | 0.00 | 0.00 | *** | Male- 66-75 (years) | HDL-AST/ALT | 0.23 | 0.00 | 0.00 | *** |
| Male- >75 (years) | VLDL-ALT/AST | 0.24 | 0.00 | 0.00 | *** | Male- >75 (years) | HDL-AST/ALT | 0.12 | 0.00 | 0.08 |  |
| Male- 18-25 (years) | VLDL-ALT | 0.33 | 0.00 | 0.00 | *** | Male- 18-25 (years) | APOB100-AST/ALT | -0.37 | 0.00 | 0.00 | *** |
| Male- 26-35 (years) | VLDL-ALT | 0.29 | 0.00 | 0.00 | *** | Male- 26-35 (years) | APOB100-AST/ALT | -0.31 | 0.00 | 0.00 | *** |
| Male- 36-45 (years) | VLDL-ALT | 0.21 | 0.00 | 0.00 | *** | Male- 36-45 (years) | APOB100-AST/ALT | -0.21 | 0.00 | 0.00 | *** |
| Male- 46-55 (years) | VLDL-ALT | 0.18 | 0.00 | 0.00 | *** | Male- 46-55 (years) | APOB100-AST/ALT | -0.13 | 0.00 | 0.00 | *** |
| Male- 56-65 (years) | VLDL-ALT | 0.18 | 0.00 | 0.00 | *** | Male- 56-65 (years) | APOB100-AST/ALT | -0.10 | 0.00 | 0.00 | *** |
| Male- 66-75 (years) | VLDL-ALT | 0.14 | 0.00 | 0.00 | *** | Male- 66-75 (years) | APOB100-AST/ALT | -0.02 | 0.34 | 1.00 |  |
| Male- >75 (years) | VLDL-ALT | 0.15 | 0.00 | 0.00 | *** | Male- >75 (years) | APOB100-AST/ALT | -0.03 | 0.38 | 1.00 |  |
| Male- 18-25 (years) | TG-UA | 0.28 | 0.00 | 0.00 | *** | Male- 18-25 (years) | APOB100-ALT/AST | 0.33 | 0.00 | 0.00 | *** |
| Male- 26-35 (years) | TG-UA | 0.28 | 0.00 | 0.00 | *** | Male- 26-35 (years) | APOB100-ALT/AST | 0.27 | 0.00 | 0.00 | *** |
| Male- 36-45 (years) | TG-UA | 0.22 | 0.00 | 0.00 | *** | Male- 36-45 (years) | APOB100-ALT/AST | 0.19 | 0.00 | 0.00 | *** |
| Male- 46-55 (years) | TG-UA | 0.20 | 0.00 | 0.00 | *** | Male- 46-55 (years) | APOB100-ALT/AST | 0.12 | 0.00 | 0.00 | *** |
| Male- 56-65 (years) | TG-UA | 0.25 | 0.00 | 0.00 | *** | Male- 56-65 (years) | APOB100-ALT/AST | 0.10 | 0.00 | 0.00 | *** |
| Male- 66-75 (years) | TG-UA | 0.22 | 0.00 | 0.00 | *** | Male- 66-75 (years) | APOB100-ALT/AST | 0.04 | 0.09 | 1.00 |  |
| Male- >75 (years) | TG-UA | 0.30 | 0.00 | 0.00 | *** | Male- >75 (years) | APOB100-ALT/AST | 0.06 | 0.06 | 1.00 |  |
| Male- 18-25 (years) | TG-AST/ALT | -0.39 | 0.00 | 0.00 | *** | Male- 18-25 (years) | APOB100-ALT | 0.31 | 0.00 | 0.00 | *** |
| Male- 26-35 (years) | TG-AST/ALT | -0.35 | 0.00 | 0.00 | *** | Male- 26-35 (years) | APOB100-ALT | 0.23 | 0.00 | 0.00 | *** |
| Male- 36-45 (years) | TG-AST/ALT | -0.27 | 0.00 | 0.00 | *** | Male- 36-45 (years) | APOB100-ALT | 0.16 | 0.00 | 0.00 | *** |
| Male- 46-55 (years) | TG-AST/ALT | -0.22 | 0.00 | 0.00 | *** | Male- 46-55 (years) | APOB100-ALT | 0.09 | 0.00 | 0.00 | *** |
| Male- 56-65 (years) | TG-AST/ALT | -0.24 | 0.00 | 0.00 | *** | Male- 56-65 (years) | APOB100-ALT | 0.05 | 0.00 | 0.05 | * |
| Male- 66-75 (years) | TG-AST/ALT | -0.20 | 0.00 | 0.00 | *** | Male- 66-75 (years) | APOB100-ALT | 0.01 | 0.72 | 1.00 |  |
| Male- >75 (years) | TG-AST/ALT | -0.16 | 0.00 | 0.00 | *** | Male- >75 (years) | APOB100-ALT | 0.00 | 0.96 | 1.00 |  |

R: Spearman correlation coefficient; Original P: original P value; Correction P: Bonferroni-corrected P value. * adjusted P<0.05; ** adjusted P<0.01; *** adjusted P<0.001.

TG: triglyceride; UA: uric acid; VLDL: very low-density lipoprotein cholesterol; ApoB100: apolipoprotein B100; HDL: high-density lipoprotein cholesterol; ALTAST: alanine aminotransferase/aspartate aminotransferase ratio; ASTALT: aspartate aminotransferase/alanine aminotransferase ratio; ALT: alanine aminotransferase.
